# Diet-induced adipose tissue expansion is mitigated in mice with a targeted inactivation of mesoderm specific transcript (*Mest*)

**DOI:** 10.1371/journal.pone.0179879

**Published:** 2017-06-22

**Authors:** Rea P. Anunciado-Koza, Justin Manuel, Randall L. Mynatt, Jingying Zhang, Leslie P. Kozak, Robert A. Koza

**Affiliations:** 1 Center for Molecular Medicine, Maine Medical Center Research Institute, Scarborough, Maine, United States of America; 2 Transgenics Core Facility, Pennington Biomedical Research Center, LSU System, Baton Rouge, Louisiana, United States of America; 3 Institute of Animal Reproduction and Food Research, Polish Academy of Sciences, Olsztyn, Poland; Vall d'Hebron Institut de Recerca, SPAIN

## Abstract

Interindividual variation of white adipose tissue (WAT) expression of mesoderm specific transcript (*Mest*), a paternally-expressed imprinted gene belonging to the α/β-hydrolase fold protein family, becomes apparent among genetically inbred mice fed high fat diet (HFD) and is positively associated with adipose tissue expansion (ATE). To elucidate a role for MEST in ATE, mice were developed with global and adipose tissue inactivation of *Mest*. Mice with homozygous (*Mest*^**gKO**^) and paternal allelic (*Mest*^**pKO**^) inactivation of *Mest* were born at expected Mendelian frequencies, showed no behavioral or physical abnormalities, and did not perturb expression of the *Mest* locus-derived microRNA miR-335. *Mest*^**pKO**^ mice fed HFD showed reduced ATE and adipocyte hypertrophy, improved glucose tolerance, and reduced WAT expression of genes associated with hypoxia and inflammation compared to littermate controls. Remarkably, caloric intake and energy expenditure were unchanged between genotypes. Mice with adipose tissue inactivation of *Mest* were phenotypically similar to *Mest*^**pKO**^, supporting a role for WAT MEST in ATE. Global profiling of WAT gene expression of HFD-fed control and *Mest*^**pKO**^ mice detected few differences between genotypes; nevertheless, genes with reduced expression in *Mest*^**pKO**^ mice were associated with immune processes and consistent with improved glucose homeostasis. Ear-derived mesenchymal stem cells (EMSC) from *Mest*^**gKO**^ mice showed no differences in adipogenic differentiation compared to control cells unless challenged by shRNA knockdown of *Gpat4*, an enzyme that mediates lipid accumulation in adipocytes. Reduced adipogenic capacity of EMSC from *Mest*^**gKO**^ after *Gpat4* knockdown suggests that MEST facilitates lipid accumulation in adipocytes. Our data suggests that reduced diet-induced ATE in MEST-deficient mice diminishes hypoxia and inflammation in WAT leading to improved glucose tolerance and insulin sensitivity. Since inactivation of *Mest* in mice has minimal additional effects aside from reduction of ATE, an intervention that mitigates MEST function in adipocytes is a plausible strategy to obviate obesity and type-2-diabetes.

## Introduction

The global rise in obesity rates are caused by interactions between an individual’s genetic predisposition, the environment and epigenetic factors. The genetic component has been clearly demonstrated in studies of monozygotic twins with heredity predicted to account for ~40–70% of obesity [1, 2]. However, recent genome wide association studies (GWAS) of 97 body mass index (BMI)-associated loci in >300K individuals was shown to account for only <3% of the variance of BMI [3]. Although it is estimated that risk alleles may account for up to ~20% of this variance [3], a large percentage of BMI is unexplained which underscores the complexity of interactions between genomic, environmental and epigenetic components [4–6]. Therefore, the use of murine models in which the genetic background and environment can be carefully controlled is a powerful tool for the identification of potential epigenetic determinants associated with the development of obesity and related disease.

Variable development of diet-induced obesity (DIO) and type 2-diabetes (T2D) within inbred populations of mice has been well described [7–11] and shown to be stable among individual animals which suggests that an epigenetic mechanism is likely involved in its etiology [10, 12, 13]. Analyses of gene expression in white adipose tissue (WAT) of inbred C57BL/6J mice that showed diversity in DIO identified a novel set of genes that correlate with fat mass accretion [10, 13–16]. Mesoderm specific transcript (*Mest*), a maternally imprinted gene, showed variations of mRNA up to ~80-fold in WAT from individual mice fed an obesogenic diet which was positively associated with the rate of fat mass deposition [10, 16]. In addition, *Mest* mRNA in WAT biopsies prior to feeding mice an obesogenic diet was predictive of future inter-individual development of DIO [10]. Compelling evidence for a role for *Mest* in facilitating fat accumulation was obtained by transgenic overexpression of *Mest* in cell culture and in mouse adipose tissue; and, in the analyses of *Mest* gene and protein in WAT during developmental growth and in adult mice fed dietary fat [7, 10, 13, 14, 16–20]. Recent studies have determined that adipose tissue as well as blood levels of *Mest* mRNA is a rapid and early biological indicator of adipose tissue expansion (ATE) in mice [21]. ATE, or fat mass expansion, is a characteristic of obesity caused predominantly by triglyceride storage and expansion of adipocytes (i.e. adipocyte hypertrophy), and to a lesser extent, increased adipocyte hyperplasia [22, 23].

The positive association of *Mest* with ATE has been well established; however, little is known in regards to the catalytic function of MEST and whether its activity directly contributes to adipocyte hypertrophy and/or adipose tissue remodeling. MEST belongs to the α/β-hydrolase family of proteins [24, 25] and contains the catalytic triad serine-histidine-aspartate (amino acids 145–147) which is commonly associated with serine proteases, lipases and acyltransferases [24, 26]. Evidence showing that MEST is localized within the endoplasmic reticulum/Golgi apparatus of the adipocyte further supports its function in the facilitation of fat storage in adipocytes [16, 20, 27]. Alternatively, MEST contains a conserved epoxide-coordinating tyrosine in its sequence and shows sequence similarity to epoxide hydrolases from *M*. *tuberculosis* [28, 29]. Epoxide hydrolases modulate levels of epoxy fatty acids and their diol derivatives which have been shown to act as endogenous mediators of PPARs alpha and gamma [30–34]. Studies have shown that epoxyeicosatrienoic acid (EET) analog treatment suppresses adipogenesis of 3T3-L1 preadipocytes and reduces ATE and improves glucose tolerance in mice fed a high fat diet [35]. It has been suggested that mitigation of adipogenic differentiation by EET may occur via Pgc1α-dependent activation of the heme oxygenase-1 (*Hmox1*)-pAKT signaling pathway [36, 37]. Other studies suggest that *Mest* may act to attenuate Wnt signaling in 3T3-L1 cells by inhibiting the maturation and exit of LRP6 from the endoplasmic reticulum by controlling its glycosylated state [27].

In adult mice, MEST protein is highly expressed in white adipose tissue with very little or undetectable expression observed in brown adipose tissue, whole brain, liver, heart, kidney and spleen [16]. Recent studies suggest that human MEST could be an epigenetic target for periconceptual or intrauterine environmental exposures, such as parental obesity and gestational diabetes, leading to changes in its methylated state [38, 39]. Although few studies have examined MEST with respect to human adipose tissue, it has been shown that MEST is induced during adipogenesis of isolated human adipocytes [40, 41] and human abdominal subcutaneous white adipose tissue biopsies show increased MEST mRNA in obese compared to non-obese individuals [41].

In this study we generated isogenic mouse models with both global and conditional targeted inactivation of *Mest* to investigate its role in adipose tissue development and expansion. Our data suggests that MEST contributes to ATE by facilitating lipid accumulation in adipocytes in an obesogenic environment thereby enhancing hypoxia and inflammation which lead to impaired glucose tolerance and insulin sensitivity.

## Results

### Validation of gene targeted *Mest* knockout mice

**Fig 1:**
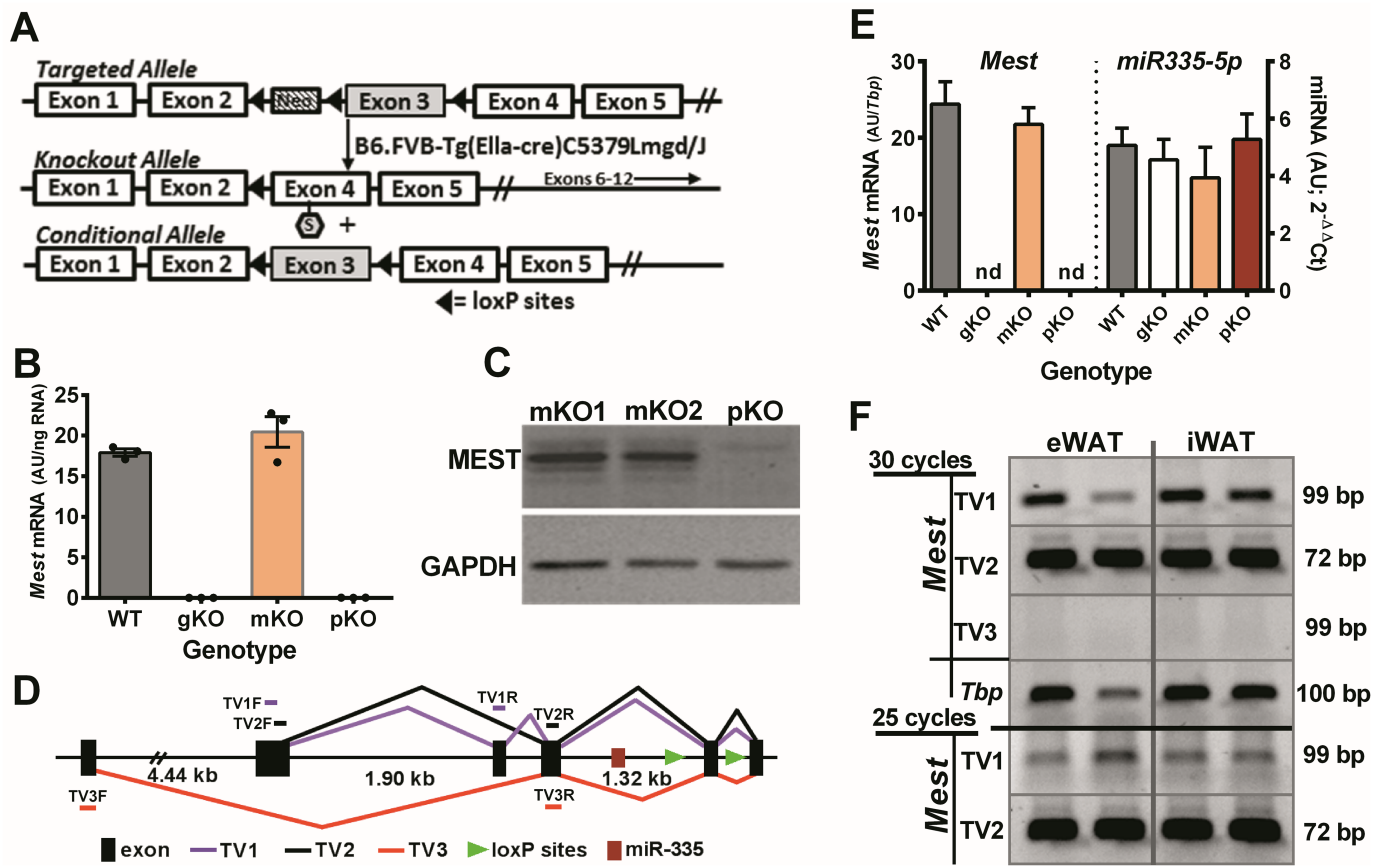
Validation of targeted *Mest* allele. (A) Representation of the targeted allele for mesoderm specific transcript (*Mest*) with *lox*P sites flanking *Mest* exon 3 and a Neo cassette located in intron 2. Deletion of *Mest* exon 3 causes a frameshift mutation and the generation of a stop codon (indicated with a hexagon containing an ‘S’) in *Mest* exon 4. (B) Analyses of *Mest* mRNA in adipose tissue of wildtype (WT; n = 3) mice and mice with homozygous (gKO; n = 3), maternal (mKO; n = 3) and paternal (pKO; n = 3) allelic inactivation of *Mest* by qRT-PCR showed complete loss of mRNA in adipose tissue of *Mest* gKO and pKO mice. (C) Western blot analysis shows MEST protein in adipose tissue of 2 mice with maternal allele inactivation of *Mest* (mKO1 and mKO2) whereas MEST protein is completely absent in adipose tissue of mice with a paternal allele inactivation of *Mest* (pKO). (D) Representation of the Mest gene showing location of *miR-335* near the proximal *lox*P site of the targeted *Mest* allele and 3 known splice variants (TV1, TV2 and TV3) for murine *Mest*. Small bars located above and below the diagram indicate the approximate location of forward reverse primers used to measure relative levels of each variant in adipose tissue. (E) QRT-PCR analyses of *Mest* and *miR-335* expression in total RNA isolated from 4–7 iWAT depots for each of 4 genotypes of 6–7 day old mice indicates that deletion of *Mest* exon 3 has minimal effect on *miR-335* expression. Levels of *Mest* and miR-335 expression are presented as the mean ± SEM. (F) RT-PCR analyses for 30 and 25 cycles indicates that transcript variant 2 (TV2; NM_008590.2) is the predominant *Mest* isoform in both epididymal white adipose tissue (eWAT) and inguinal white adipose tissue (iWAT). Sizes of PCR products for each of the transcript variants and TATA-box binding protein (*Tbp*) are shown on the right hand side of the figure.

A targeting vector containing a neomycin (NEO) selection cassette was used to insert loxP sites immediately 5’ and 3’ of *Mest* exon 3 using C57BL/6J (B6) ES cells (Fig 1A). Subsequent breeding of mice with germline transmission of the targeted *Mest* allele with *Ella*-cre transgenic mice resulted in the selection of progeny with deletions of only the NEO cassette or the floxed region (NEO cassette and *Mest* exon 3) resulting in an intact floxed or a global deletion of *Mest* exon 3. Deletion of exon 3 of *Mest* creates a stop codon in exon 4 and full inactivation of *Mest* as shown by the absence of *Mest* mRNA in white adipose tissue (WAT) of homozygous knockout (*Mest*^**gKO**^) mice (Fig 1B). *Mest*, an imprinted gene that is almost exclusively expressed from the paternal allele in adult mice [42–45] and highly expressed in WAT [10, 16, 46], is completely abrogated in mice with a paternal allelic inactivation of *Mest* (*Mest*^**pKO**^), but shows fully intact mRNA and protein levels when the maternal allele of *Mest* is selectively inactivated (*Mest*^**mKO**^; Fig 1B and 1C). Unlike previous observations showing abnormal maternal behavior and high incidence of postnatal mortality in mice with global or paternal inactivation of *Mest* on a mixed B6/129 background (Lefebvre et al 1998), our studies indicate normal litter sizes reared by dams with inactivated *Mest* (~8.2 pups/litter); and non-skewed gender (29 female/30 male) and genotype ratios with 14 wildtype (WT), 30 *Mest*^**mKO**^ or *Mest*^**pKO**^ and 15 *Mest*^**gKO**^ (chi-square 0.119; P = 0.998) born from crosses between heterozygous females and males. The basis for phenotypic differences in maternal behavior and early post-natal mortality between our studies and those of Lefebvre et al 1998 [43] are not known.

Since the *Mest* locus harbors a microRNA (miR-335), which has also been implicated in adipogenesis and lipid metabolism [47, 48], within an intronic sequence between exons 2 and 3 of *Mest* transcript variant 2 (TV2), an analyses was performed to determine whether the close proximity of a loxP site (Fig 1D) from the targeting vector aberrantly affects expression of miR-335. Analyses of inguinal (iWAT) WAT of 6–7 day old mice, a developmental time period when fat mass is rapidly expanding and WAT *Mest* is highly expressed [16], showed no differences in miR-335 expression in mice with intact (WT and *Mest*^**mKO**^) versus inactivated (*Mest*^**gKO**^ and *Mest*^**pKO**^) *Mest* (Fig 1E). In addition, RT-PCR analyses of 3 known transcriptional variants of *Mest* in mice that vary in the use of alternative exons (Fig 1D) shows that transcript variant 2 (TV2; NM_008590.2) is predominantly expressed in both subcutaneous iWAT and visceral epididymal (eWAT) WAT depots (Fig 1F). *Mest* TV1, which uses an additional alternative exon in the 5’ coding region compared to TV2, also shows a low level of expression in WAT, whereas TV3, a variant of *Mest* mRNA that uses an alternative 5’UTR ~4.5 kb upstream from the 5’UTR containing exon of TV1 and TV2, was absent in WAT (Fig 1F).

### Bodyweight and body composition

**Fig 2:**
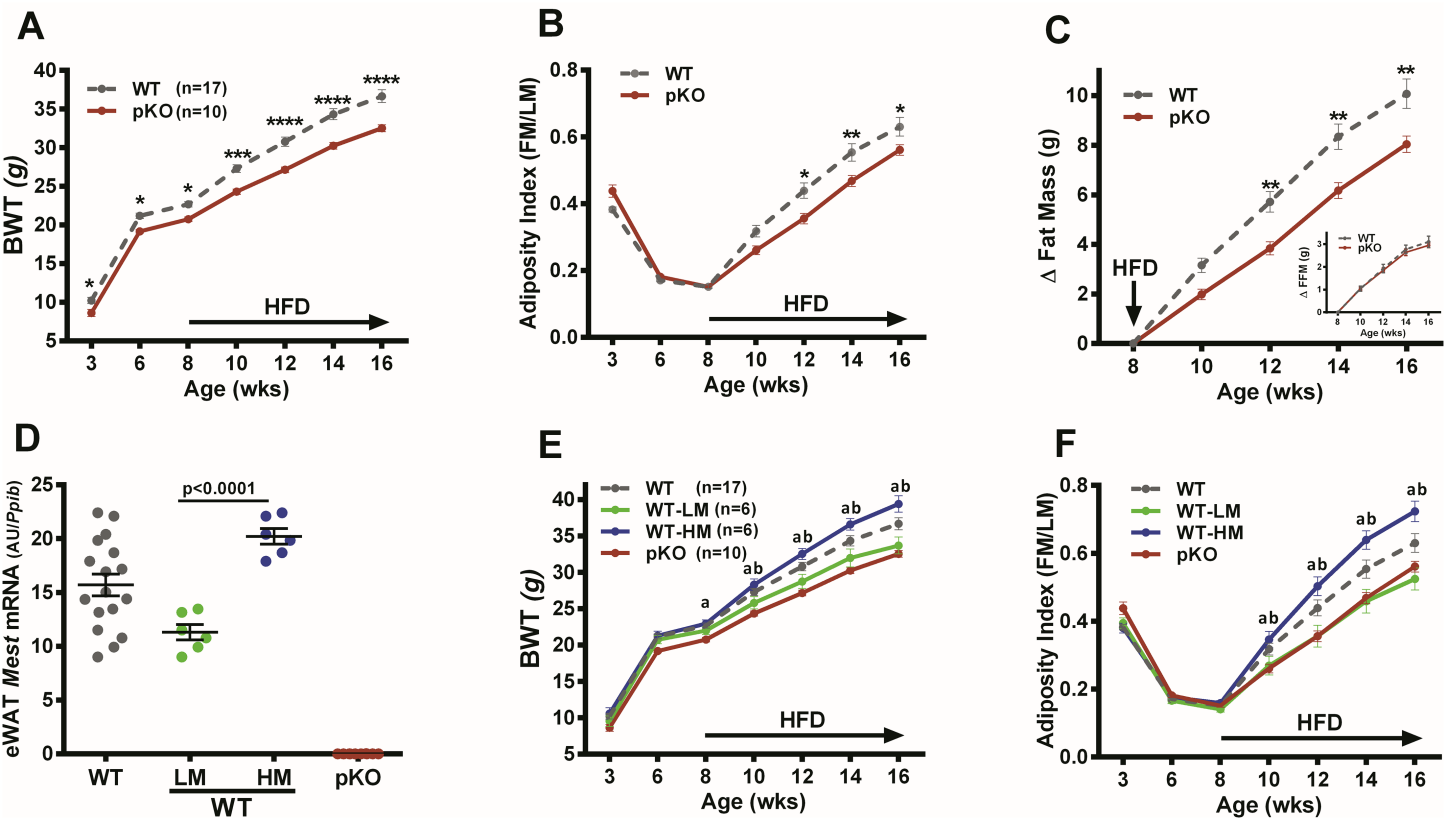
Dietary obesity in mice with inactivated *Mest*. (A) Longitudinal measurement of bodyweight (BWT) in 17 wildtype (WT) mice and 10 mice with inactivation of *Mest* on the paternal allele (pKO). Mice were fed high fat diet (HFD) from 8 to 16 weeks of age as indicated by the arrow along the X-axis. (B) Longitudinal measurements show the ratio of fat mass (FM) and lean mass (LM) measured by NMR, indicated as adiposity index (FM/LM), in 17 WT and 10 pKO mice fed a HFD from 8 to 16 weeks as shown by the arrow along the X-axis. (C) Data shows the cumulative change of fat mass (Δ Fat Mass) and fat-free mass (Δ FFM; inset graph) of 17 WT and 10 *Mest*^**pKO**^ (pKO) mice after initiation of a HFD at 8 weeks of age until 16 weeks of age as indicated on the X-axis. (D) Data represents tertiles of mice from the 17 WT mice in the study with the lowest (WT-LM; n = 6) and highest (WT-HM; n = 6) epididymal white adipose tissue (eWAT) *Mest* expression. Gene expression was measured by TaqMan QRT-PCR and is represented as arbitrary units (AU) normalized to cyclophilin b (*Ppib*). (E) Longitudinal analyses of BWT and (F) adiposity index in mice within WT-LM and WT-HM tertiles for eWAT *Mest* and pKO mice are shown. Data in all figures are presented as the mean ± SEM. Significance at each time point of the longitudinal phenotypic analyses between WT and pKO (A- C) was determined by 2-way ANOVA using the Holm-Sidak multiple comparisons test. Time points annotated with 1, 2, 3 or 4 asterisks indicate significant differences of P≤0.05, P≤0.01, P≤0.001 and P≤0.0001 respectively. Significance in eWAT *Mest* RNA expression between the WT-LM and WT-HM groups (D) was measured by a two-tailed unpaired parametric t-test and is indicated on the bar graph. Longitudinal data for WT-LM, WT-HM and pKO (E and F) was performed using 2-way ANOVA using Tukey’s multiple comparisons test. Annotation with ‘a’ and ‘b’ indicates significant differences between WT-HM vs pKO and WT-HM vs WT-LM mice respectively.

Since *Mest*^**pKO**^ mice do not express *Mest* mRNA or protein in WAT (Fig 1B and 1C), male progeny (WT and *Mest*^**pKO**^) for the dietary obesity study were generated from a cross between female WT and male *Mest*^**mKO**^ mice. From weaning (week 3) until 8 weeks of age, *Mest*^**pKO**^ mice showed a small but significant reduction in body weight (Fig 2A) but no differences in adiposity measured as the ratio of fat mass/lean mass (Fig 2B) compared to WT littermate controls. However, when fed an obesogenic diet (58 kcal% fat) from 8 until 16 weeks of age, dietary fat-induced increases in bodyweight, adiposity and fat mass deposition in *Mest*^**pKO**^ mice was significantly attenuated compared to WT mice (Fig 2A, 2B and 2C) whereas fat free mass (FFM) was unaffected (Fig 2C; inset). Regression analyses of the 17 WT mice at 16 weeks of age showed a strong correlation between eWAT *Mest* mRNA and fat mass deposition (R = 0.70; p<0.002) as well as *Mest* mRNA expression among adipose depots (iWAT vs eWAT *Mest*; R = 0.59; P<0.02) within each mouse which is consistent with previous studies [10, 16]. Analyses of subgroups of WT mice within the lowest (n = 6; WT-LM) and highest (n = 6; WT-HM) tertiles for eWAT *Mest* mRNA (Fig 2D) showed significant differences in BWT and adiposity between groups (Fig 2E and 2F). However, no differences were observed between WT-LM and *Mest*^**pKO**^ mice (Fig 2E and 2F). These data suggest that the low level of MEST in adipose tissue of WT-LM mice does not significantly contribute to additional ATE beyond that observed for *Mest*^**pKO**^ mice. Thus, increased ATE only becomes apparent in mice that show high interindividual WAT *Mest* expression.

### Adipose tissue inactivation of *Mest*

**Fig 3:**
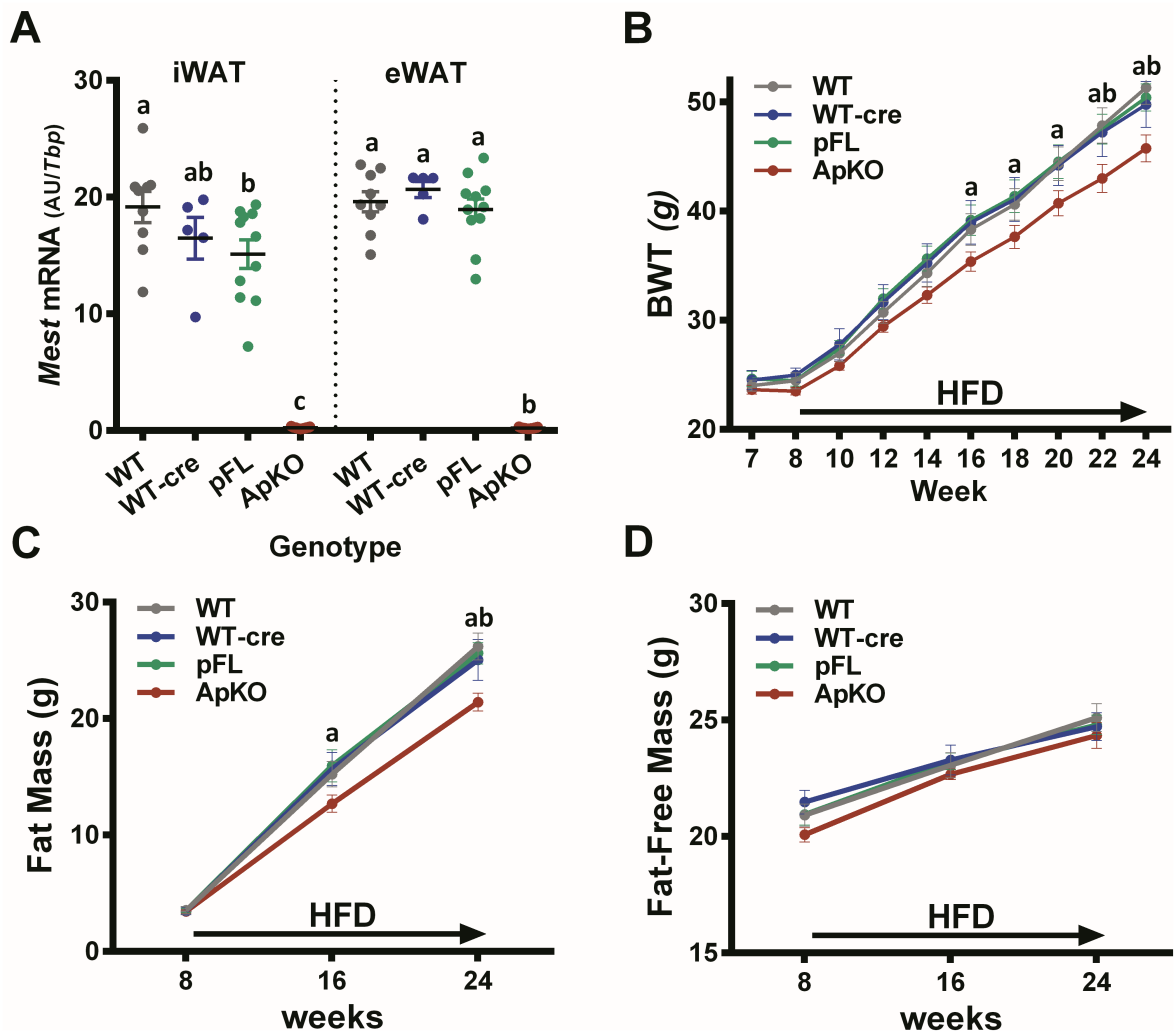
Dietary obesity in mice with *Adipoq*-cre mediated inactivation of *Mest*. (A) *Mest* mRNA expression in inguinal (iWAT) and epididymal (eWAT) white adipose tissue of wildtype (WT; n = 9), WT.*Adipoq*-cre (WT-cre; n = 5), paternal floxed *Mest* (pFL; n = 11) and pFL.*Adipoq*-cre (ApKO; n = 17) mice after being fed a HFD from 8–24 weeks of age. *Mest* mRNA expression measured by TaqMan QRT-PCR is represented as the mean ± SEM of arbitrary units (AU) normalized to TATA-box binding protein (*Tbp*). Significance in *Mest* RNA expression between groups was determined via one-way ANOVA using Tukey’s multiple comparisons test. (B) Data shows the longitudinal measurements of bodyweight (BWT) for WT (n = 9), WT-cre (n = 5), pFL (n = 11) and ApKO (n = 17) mice fed a HFD from 8 to 24 weeks of age as indicated by the arrow along the X-axis. (C) Longitudinal measurements of fat mass (g) and (D) fat-free mass (g) measured by DEXA at the times indicated on the X-axis are shown for WT (n = 9), WT-cre (n = 5), pFL (n = 11) and ApKO (n = 17) mice fed a HFD from 8 to 24 weeks of age as indicated by the arrow along the X-axis. (B-D) All data in the longitudinal studies are presented as the mean ± SEM. Significance at each time point of the longitudinal phenotypic analyses was determined by 2-way ANOVA and Tukey’s multiple comparisons test. Time points annotated with ‘a’ and ‘b’ indicates significant differences between ‘ApKO vs pFL’ and ‘ApKO vs WT’.

Mice with a floxed paternal allele for *Mest (Mest*^**pFL**^; Fig 1A*)* were crossed with *Adipoq*-cre transgenic mice to generate animals with an *Adipoq*-cre mediated inactivation of the paternal allele (*Mest*^**ApKO**^) of *Mest*. Analyses of WAT *Mes*t mRNA in cohorts of 5–17 age-matched male mice fed an obesogenic diet for 16 weeks showed a >98% reduction in both iWAT and eWAT *Mest* mRNA expression (Fig 3A) in *Mest*^**ApKO**^ mice compared with the 3 control groups (i.e. WT, WT-cre and *Mest*^**pFL**^; Fig 3A) and supports previous studies showing that *Mest* is expressed in the mature adipocyte fraction of adipocytes [10, 20]. In addition, the presence of *loxP* sites in the 5’ and 3’ introns surrounding *Mest* exon 3 of *Mest*^**pFL**^ did not adversely affect *Mest* expression. Longitudinal analyses of bodyweight and body composition in mice fed a HFD shows significantly reduced bodyweight gain and fat mass accumulation in the *Mest*^**ApKO**^ mice (Fig 3B and 3C), but no changes in fat-free mass (Fig 3D), compared to the 3 control groups. Reduced bodyweight and adiposity in *Mest*^**ApKO**^ mice is consistent with the phenotypes observed for the *Mest*^**pKO**^ mice (Fig 2). Both genetic models suggest that MEST facilitates ATE in an obesogenic environment. An additional cohort of mice with *Fabp4*-cre mediated inactivation of *Mest* show similar phenotypic profiles compared to the *Mest*^**ApKO**^ mice (S1 Fig).

### Metabolic parameters and adipocyte morphology

**Fig 4:**
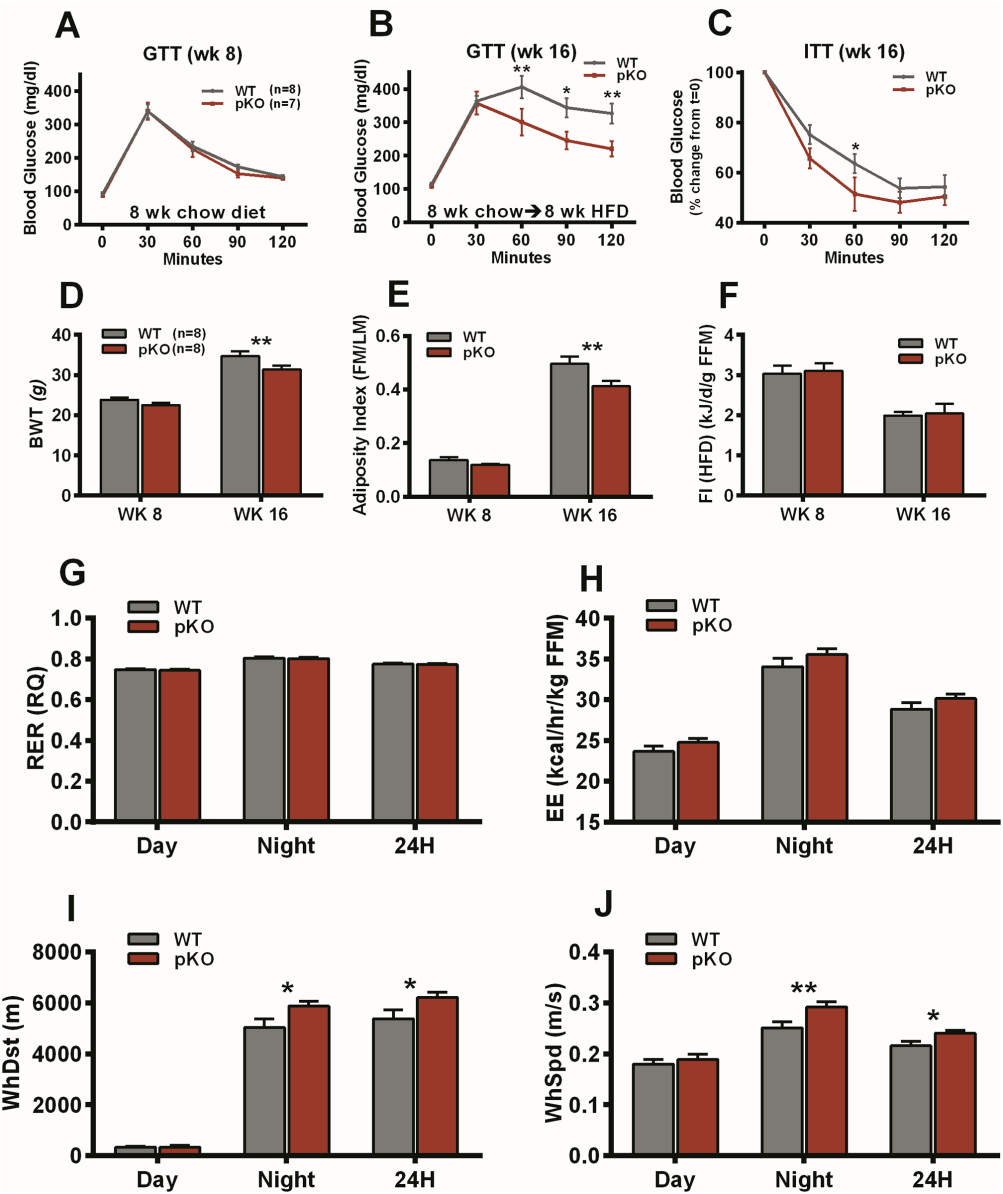
Diabetic and metabolic measurements of mice with inactivated *Mest*. (A) Data shows glucose tolerance test (GTT) of WT (n = 8) and *Mest*^**pKO**^ (pKO) mice (n = 7) fed a chow diet at 8 weeks of age. (B) GTT and (C) insulin tolerance test (ITT) of WT (n = 8) and pKO (n = 7) at 16 weeks of age after feeding mice a high fat diet (HFD) for 8 weeks. (D) Bodyweight (BWT), (E) adiposity index (fat mass/lean mass; FM/LM) and (F) food intake (FI; kJ/day normalized to fat-free mass (FFM) are presented for an additional cohort of WT (n = 8) and pKO (n = 8) mice fed a HFD from 8 to 16 weeks of age that were subjected to indirect calorimetric analyses. Body composition measurements were performed via DEXA. Indirect calorimetric measurements of (G) respiratory quotient (RQ), (H) energy expenditure (EE), (I) wheel running distance (WhDst) and (J) wheel speed (WhSpd) were performed on mice for 1 week after being fed a HFD for 8 weeks. All data is presented as the mean ± SEM. Significance between groups was determined using two-tailed unpaired parametric t-tests and 1 or 2 asterisks indicate significant P-values of ≤0.05 and ≤0.01 respectively.

To prevent aberrant phenotypic measurements due to stress from over-manipulation of the animals, two additional cohorts of *Mest*^**pKO**^ mice and littermate WT controls were used to examine diabetic and metabolic parameters. A cohort used to measure glucose (GTT) and insulin tolerance (ITT) showed no significant differences in bodyweight (WT, N = 8, 22.51 ± 0.26 g vs *Mest*^**pKO**^, N = 7, 22.13 ± 0.30 g; p = 0.35) or glucose tolerance (Fig 4A) between chow-fed WT and *Mest*^**pKO**^ mice at 8 weeks of age. However, after feeding mice a HFD from 8 to 16 weeks of age, *Mest*^**pKO**^ mice showed reduced bodyweight (WT, 38.46 ± 0.96 grams vs *Mest*^**pKO**^, 33.71 ± 1.45 grams; p<0.02) and improved glucose (Fig 4B) and insulin (Fig 4C) tolerance compared to WT littermate controls.

An independent cohort of HFD-fed WT (n = 8) and *Mest*^**pKO**^ (n = 8) mice that were subjected to indirect calorimetry showed similar differences in both bodyweight and adiposity (Fig 4D and 4E) as previously observed. Surprisingly, caloric intake normalized to fat-free mass (kJ/d/g FFM) measured in mice fed HFD at 8 and 16 weeks of age was similar between WT and *Mest*^**pKO**^ mice (Fig 4F) suggesting that differences in food consumption is not a causative factor for the divergence in adiposity between genotypes. Respiratory quotient (RQ) measured in 16 week old mice after feeding a HFD for 8 weeks were identical between genotypes (Fig 4G) and RQ values levels were consistently below 0.8 indicating that fat, as expected due to feeding the mice HFD, is the primary fuel source for both genotypes. Although no significant differences in energy expenditure (EE; kcal/hr/kg FFM) were noted between genotypes (Fig 4H) at 16 weeks of age, *Mest*^**pKO**^ mice did show a ~4–5% elevation in 24 hour EE compared to WT mice. Analyses of physical activity based on wheel running at 16 weeks of age shows that *Mest*^**pKO**^ mice consistently ran ~15% further (Fig 4I; p≤0.05) and faster (Fig 4J; p≤0.01) than WT mice at night which could at least partially explain the slight increase in EE observed for *Mest*^**pKO**^ mice.

**Fig 5:**
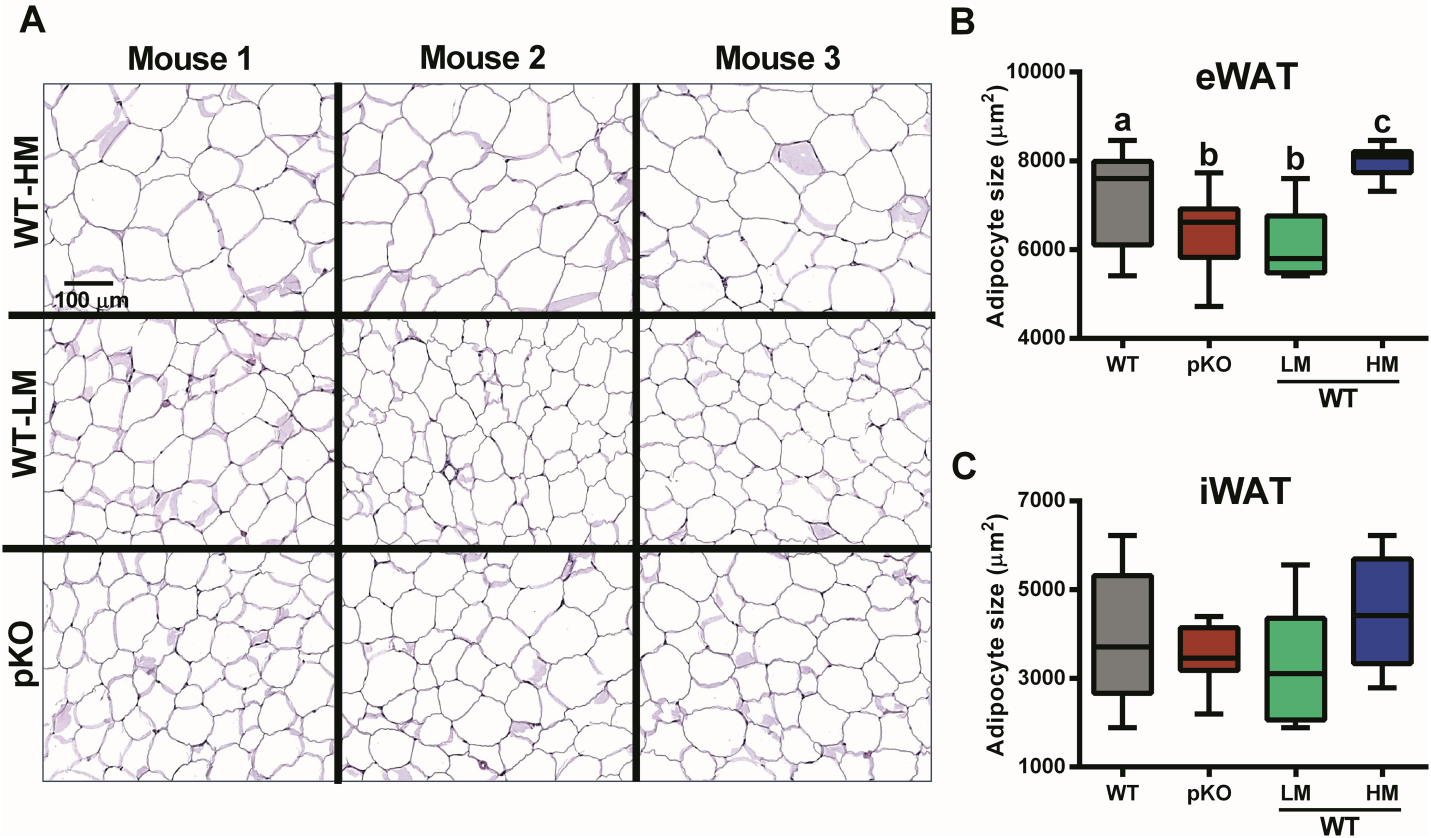
Adipocyte size determination in mice with inactivated *Mest*. (A) A representative image shows variation in the size of adipocytes in eWAT of WT mice with high (WT-HM; n = 3) and low (WT-LM; n = 3) epididymal adipose tissue (eWAT) *Mest* expression and *Mest*^**pKO**^ (pKO) mice (n = 3). (B and C) Box and whisker graphs show the median and range of adipocyte sizes determined morphometrically for WT (n = 17), pKO (n = 10), WT-LM (n = 6) and WT-HM (n = 6) mice for both eWAT and inguinal adipose tissue (iWAT). Significance between groups was determined using two-tailed unpaired parametric t-tests. Datasets annotated with the same letter indicate no significant differences between groups.

Adipocyte morphology for both eWAT and iWAT, determined for the cohort of mice used in the HFD study described in Fig 2 shows that WT mice with high WAT *Mest* expression (WT-HM) have significantly larger adipocytes in eWAT (Fig 5A and 5B) compared to *Mest*^**pKO**^ (p<0.005) or WT mice with low WAT *Mest* expression (WT-LM; p<0.001). Although the size of adipocytes in iWAT (Fig 5C) was not different between genotypes, the general trend of increased adipocyte hypertrophy in mice within the HM group was evident in both WAT depots. These results are consistent with previous studies using B6 mice fed a HFD that showed a positive correlation between adiposity and adipocyte size with WAT *Mest* mRNA and protein [16].

### Gene expression analyses

**Fig 6:**
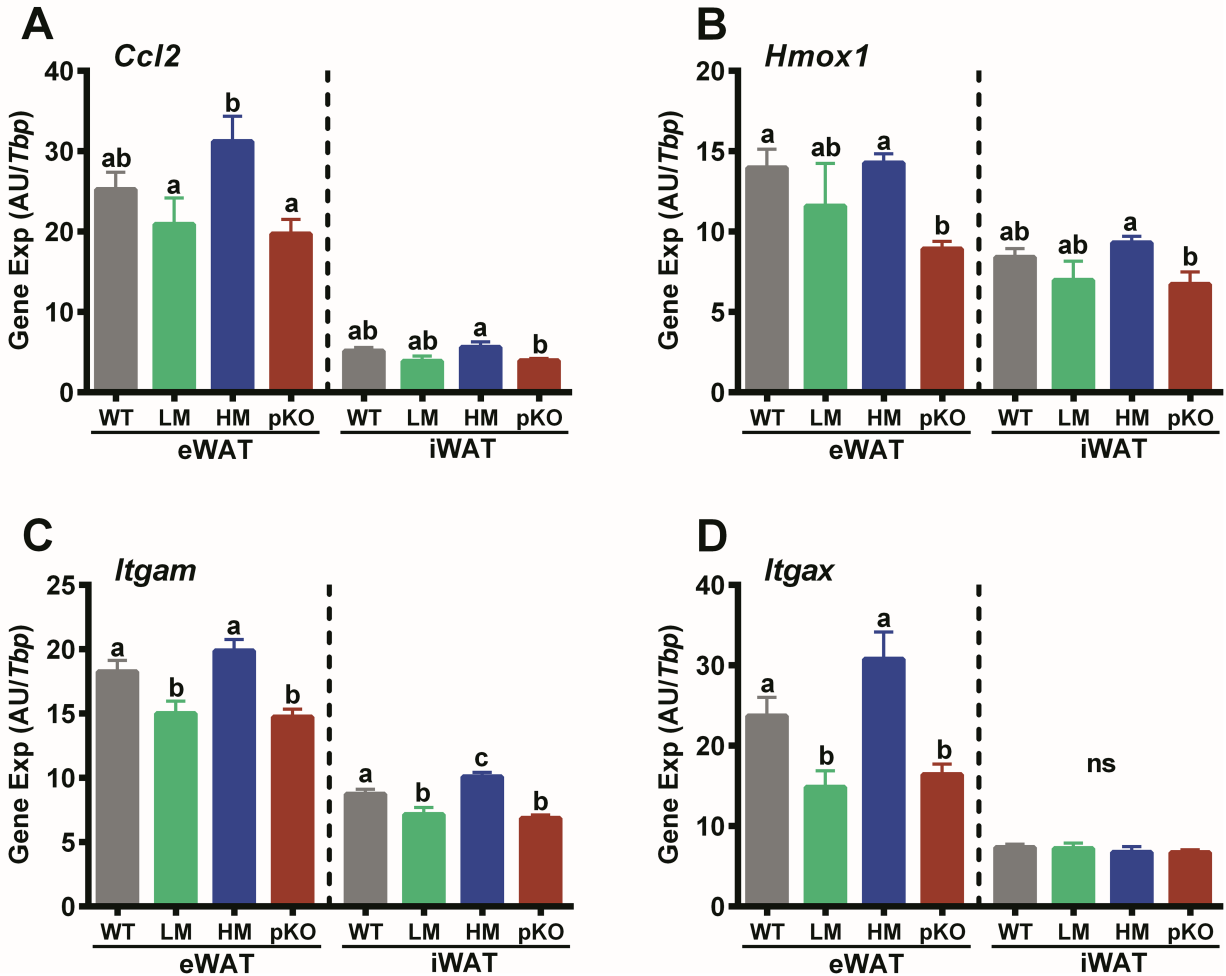
Inflammatory genes in WAT of mice with inactivated *Mest*. A, B, C and D show expression of gene markers for inflammation and macrophage infiltration in WAT of WT and *Mest*^**pKO**^ (pKO) mice. Gene expression was determined via TaqMan qRT-PCR in total RNA from eWAT and iWAT of wildtype (WT; n = 17), mice with a paternal inactivation of *Mest* (pKO; n = 10) and subgroups of WT mice with low (LM; n = 6) and high (HM; n = 6) WAT *Mest* expression after being fed a HFD from 8–16 weeks of age. Gene expression is represented as arbitrary units (AU) normalized to *Tbp* and presented as the mean ± SEM. Statistical analysis between genotypes within each WAT depot was performed using two-tailed unpaired parametric t-tests and datasets annotated with the same letter indicate no significant differences between groups.

Gene expression markers for macrophage infiltration/inflammation were used to determine whether improved glucose tolerance in HFD-fed *Mest*^**pKO**^ mice (Fig 4B) is concomitant with reduced inflammatory state of WAT. Gene expression analyses of adipose tissue of the WT, *Mest*^**pKO**^, WT-LM and WT-HM mice used in the HFD study described in Fig 2 showed reduced expression of *Ccl2* (Mcp-1), *Hmox1*, *Itgam* and *Itgax* in eWAT of *Mest*^**pKO**^ compared to the WT and WT-HM mice (Fig 6A, 6B, 6C and 6D). Although differences in the expression of these gene markers between groups were not as prominent in iWAT compared with eWAT, they were consistent with that observed for eWAT except for *Itgax* which showed no differences between groups. The absence of differences in gene expression between the WT-LM and *Mest*^**pKO**^ mice suggest that changes in gene expression in *Mest*^**pKO**^ mice are associated with the reduction of ATE and suppression of inflammation. Furthermore, the analyses of hepatic gene expression in WT vs *Mest*^**pKO**^ mice show no differences in the expression of *Fas* (WT; 10.09 ± 0.75 vs *Mest*^**pKO**^; 10.47 ± 1.53 AU/Tbp; p = 0.83) and *Scd1* (WT; 11.55 ± 0.70 vs *Mest*^**pKO**^; 11.93 ± 1.22 AU/Tbp; p = 0.79), genes involved in hepatic *de novo* lipogenesis that have been shown to be positively associated with hepatic steatosis [49–52]. These data indicate that reduced ATE in *Mest*^**pKO**^ mice does not result in the development of a lipodystrophic state characterized by excessive hepatic accumulation of lipid.

**Fig 7:**
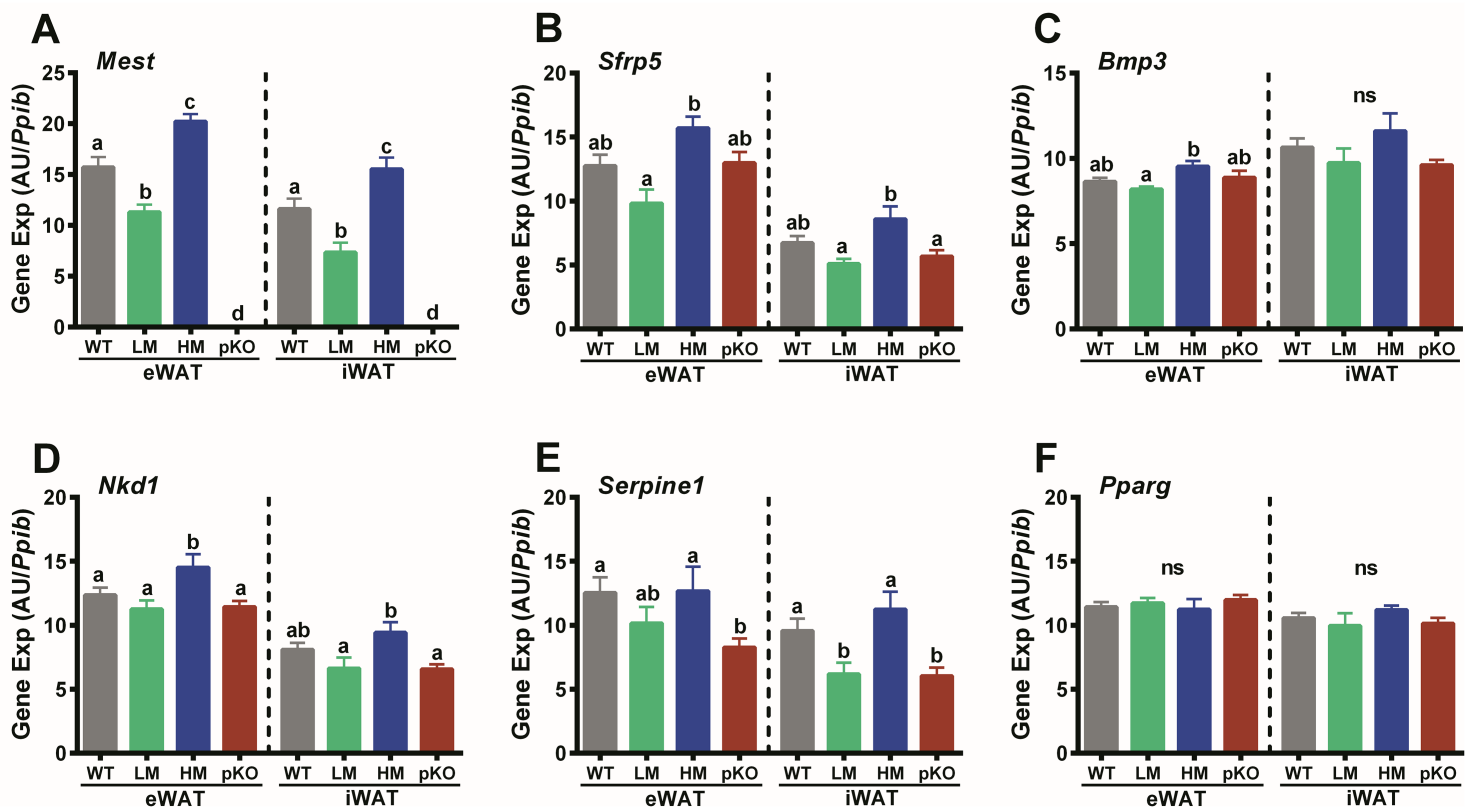
WAT gene expression in mice with inactivated *Mest*. **(A)** Data shows expression of *Mest*, (B) *Sfrp5*, (C) *Bmp3*, (D) *Nkd1*, (E) *Serpine1*, and (F) *Pparg* in epididymal (eWAT) and inguinal (iWAT) white adipose tissue of wildtype (WT; n = 17) mice with a paternal inactivation of *Mest* (pKO; n = 10) and subgroups of WT mice with low (LM; n = 6) and high (HM; n = 6) WAT *Mest* expression after being fed a HFD from 8–16 weeks of age. Gene expression was determined via TaqMan qRT-PCR in total RNA from eWAT and iWAT and represented as arbitrary units (AU) normalized to cyclophilin b (*Ppib*). Gene expression is presented as the mean ± SEM and significance between groups was determined using two-tailed unpaired parametric t-tests. Datasets annotated with the same letter indicate no significant differences between groups.

Activation of both canonical and non-canonical Wnt signaling has been shown to inhibit adipogenesis, mediate adipose tissue development and expansion and promote adipose tissue inflammation in obesogenic conditions [53–55]. Because previous studies have shown that adipose tissue *Mest* mRNA expression is positively associated with a subset of genes that include Wnt signaling inhibitors (i.e. *Sfrp5* and *Nkd1*), in addition to the *Tgfb*-pathway mediator *Bmp3* and the serine protease inhibitor *Serpine1* [10, 13, 16], an analyses of this subset of genes in eWAT and iWAT of WT and *Mest*^**pKO**^ mice from the HFD study described in Fig 2 was performed to determine effects of *Mest* inactivation on their expression. Surprisingly, inactivation of *Mest* did not significantly alter WAT expression of *Sfrp5*, *Bmp3*, *Nkd1* and *Serpine1* (Fig 7B, 7C, 7D and 7E) with levels of expression comparable to WT-LM mice. However, consistent with previous studies [10, 13, 16], WT-HM mice did show elevated levels of *Sfrp5*, *Bmp3*, *Nkd1* and *Serpine1* expression compared with WT-LM mice for both WAT depots (Fig 7B, 7C, 7D and 7E). Additional regression analyses of gene expression in WAT of the 17 WT mice used in the HFD-study in Fig 2 showed significant correlations between *Mest* vs *Sfrp5* (p = 0.00067), *Bmp3* (p = 0.013) and *Nkd1* (p = 0.031) in eWAT; and, between *Mest* vs *Sfrp5* (p = 0.000025), *Bmp3* (p = 0.013), *Nkd1* (p = 0.0081) and *Serpine1* (p = 0.0052) in iWAT. No differences in *Pparg* expression were observed between groups (Fig 7F) and *Pparg* mRNA in WAT was not associated with WAT *Mes*t as shown in previous studies [7].

**Fig 8:**
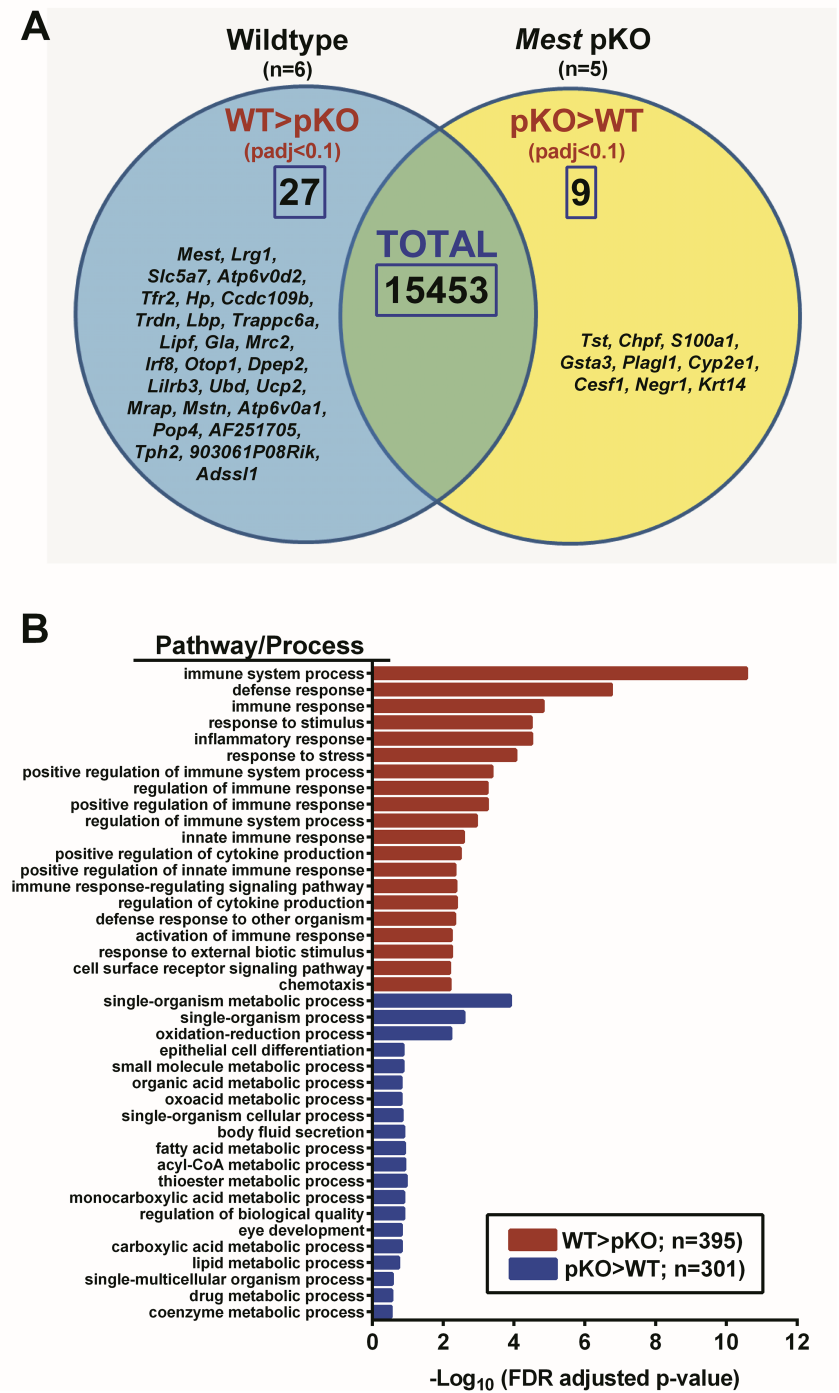
Global analyses of gene expression in eWAT of mice with global inactivation of *Mest*. (A) A Venn diagram represents a summary of ABI SoLID SAGE analyses of differentially expressed genes in eWAT of 6-wildtype (WT) and 5- *Mest*^**pKO**^ (pKO) mice fed a high fat diet from 8 to 16 weeks of age. Differentially expressed genes have a false discovery rate (FDR) adjusted P-value ≤ 0.1 and at least one genotype with ≥ 50 sequenced tags per gene. (B) The bar graph represents pathway analyses of 695 genes differentially expressed in eWAT of WT versus *Mest*^**pKO**^ mice that have non-adjusted P-values of ≤ 0.05 and at least one genotype with ≥ 50 sequenced tags per gene (S2 Table).

A global analysis of gene expression via ABI SOLiD SAGE sequencing was used to identify genes and genetic pathways that are dysregulated in adipose tissue of mice with a targeted inactivation of *Mest* (Fig 8). Gene profiles for eWAT were analyzed for 6 WT and 5 *Mest*^**pKO**^ mice selected from the cohort used in the HFD study in Fig 2. Adiposity indices (FM/LM ratio) of the WT mice were significantly higher than *Mest*^**pKO**^ mice (WT, 0.724 ± 0.030; *Mest*^**pKO**^, 0.571 ± 0.019; p = 0.003) after the 8 weeks of HFD. A Venn diagram (Fig 8A) shows surprisingly few genes from the 15,453 gene targets detected in eWAT to have significantly higher (n = 27) or lower expression (n = 9) in WT vs *Mest*^**pKO**^ mice when FDR-adjusted to P<0.1 (S1 Table). *Mest*, as expected, was significantly reduced >15-fold in eWAT of *Mest*^**pKO**^ mice and TaqMan qRT-PCR validation showed undetectable (reduced >100-fold) levels of *Mest* in eWAT and iWAT of *Mest*^**pKO**^ mice (Table 1). Analyses of 14 additional selected genes with RNA from the individual eWAT samples used for ABI SOLiD SAGE sequencing via qRT-PCR showed significant and consistent differences between WT and *Mest*^**pKO**^ mice in the expression of 10 genes whereas 2 genes (*Negr1*, *Mrap*) showed no significant differences between groups and 2 genes (*Plagl2*, *Tph2*) could not be detected (Table 1). Additional analyses of eWAT RNA from the entire cohort of 17-WT and 10- *Mest*^**pKO**^ mice also showed comparable patterns of expression for the 10 differentially-expressed genes with the global and sample validation studies (Table 1). Importantly, 7 of the 10 genes showed similar expression patterns for both iWAT and eWAT whereas the expression of 3 genes (*Gsta3*, *Ucp2* and *Ces1f*) only showed differences in eWAT.

**Table 1:** Validation of differentially-expressed genes in eWAT of WT and pKO mice.

	ABI SOLiD SAGE Analyses			qRT-PCR validation			
Gene	FC	pval^a^	padj^b^	FC(WT/pKO	pval^c^	FC (17-WT/10-pKO)	pval (ttest)^c^
	(WT/pKO)			SAGE RNA	(ttest)	RNA; eWAT/iWAT	RNA; eWAT/iWAT
***Mest***	**15.09**	**1.6E-65**	**2.5E-61**	**>100**	**3.5E-06**	**>100/>100**	**1.3E-12/3.3E-09**
***Lrg1***	**2.26**	**9.6E-09**	**4.4E-05**	**1.57**	**2.6E-04**	**1.40/1.35**	**0.0019/0.0015**
***Slc5a7***	**2.21**	**1.4E-08**	**4.4E-05**	**2.10**	**6.9E-05**	**1.79/1.65**	**2.4E-04/0.0030**
***Atp6v0d2***	**2.69**	**1.2E-08**	**4.4E-05**	**2.29**	**0.0072**	**1.86/1.83**	**0.0023/0.0014**
***Tfr2***	**2.71**	**1.2E-08**	**4.4E-05**	**2.29**	**0.00040**	**1.77/1.84**	**0.0010/0.0011**
***Gsta3***	**0.52**	**5.4E-06**	**0.0070**	**0.61**	**0.021**	**0.77/1.01**	**0.016/0.89**
***Lbp***	**1.87**	**7.2E-06**	**0.0076**	**1.42**	**0.0044**	**1.27/1.53**	**0.0013/0.0017**
***Lipf***	**2.16**	**7.3E-06**	**0.0076**	**2.57**	**0.0032**	**1.81/2.37**	**0.0020/0.0015**
***Mrc2***	**1.93**	**1.2E-05**	**0.011**	**2.12**	**0.0043**	**1.61/1.66**	**0.0032/0.0021**
***Plagl1***	**0.55**	**2.0E-05**	**0.016**	**ND**	**ND**	**ND**	**ND**
***Ucp2***	**1.67**	**8.9E-05**	**0.053**	**1.43**	**0.0082**	**1.34/1.10**	**0.0057/0.13**
***Ces1f***	**0.46**	**8.0E-05**	**0.053**	**0.52**	**0.0032**	**0.71/0.97**	**0.010/0.64**
***Negr1***	**0.58**	**1.2E-04**	**0.062**	**0.78**	**0.11**	**0.92/1.02**	**0.33/0.76**
***Mrap***	**1.71**	**1.1E-04**	**0.062**	**1.18**	**0.12**	**1.13/1.12**	**0.16/0.44**
***Tph2***	**4.52**	**2.1E-04**	**0.094**	**ND**	**ND**	**ND**	**ND**

eWAT, epididymal white adipose tissue; iWAT, inguinal white adipose tissue; WT, wildtype mice; pKO, *Mest*^**pKO**^ mice; FC, fold change; SAGE, serial analyses of gene expression; ND, not detected.

^a^p-values were calculated using SAM as described in Materials and Methods.

^b^p-values were adjusted for false discovery rate using the Benjamini-Hochberg method.

^c^p-values were determined using two-tailed unpaired parametric t-tests.

Ontology and enrichment analyses of the 27 genes with reduced expression in eWAT of *Mest*^**pKO**^ mice showed associations with several biological processes including cation transport (*Tfr2*, *Atp6v0a1*, *Atp6v0d2*, *Slc5a7*, *Ccdc109b* and *Trdn*; P<10^−4^), regulation of growth of symbiont in host (*Lbp*, *Irf8;* P<10^−3^), myeloid leukocyte activation (*Lbp*, *Pirb*, *Ubd;* P<10^−3^) and acute phase response (*Lbp*, *Hp;* P<10^−3^); however, none of the associations were significant after FDR correction. Genes more highly expressed in eWAT of *Mest*^**pKO**^ showed modest association with mycotoxin/aflatoxin catabolism and metabolism (*Gsta3;* P<10^−3^) and monoterpenoid metabolism (*Cyp2e1;* P<10^−3^). Further enrichment analyses performed on non-FDR-adjusted gene sets with a P-value cutoff of ≤ 0.05 and a minimum of 50 sequence tags for each mRNA in at least one of the groups identified 695 gene targets that were differentially expressed in eWAT of WT vs *Mest*^**pKO**^ mice (S2 Table). The 395 genes with reduced expression in eWAT of *Mest*^**pKO**^ mice showed highly significant associations with immune system processes (Padjusted <10^−10^) including defense, immune, inflammatory and stress responses whereas genes with increased expression in eWAT of *Mest*^**pKO**^ mice (n = 301) showed enrichment for single-organism metabolic processes (Padjusted<10^−3^) including oxidation-reduction, organic acid metabolism; and, fatty acid, acyl-CoA, thioester and monocarboxylic acid metabolism (Fig 8B). An examination of the expression of the mature mouse macrophage gene marker F4/80 (Emr1; *Adgre1*), and gene markers that delineate classically (M1) versus alternatively (M2) macrophage subtypes [56] only showed significantly elevated expression for the M1 macrophage marker *Gpr18* (p = 0.04) and M2 macrophage marker *Egr2* (p = 0.02) in eWAT of WT mice. No differences in expression between genotypes were observed for F4/80, *Cd38*, *Fpr2*, *Cd163*, *Cd68 and Myc*.

### Adipogenic differentiation of Ear-derived mesenchymal stem cells

**Fig 9:**
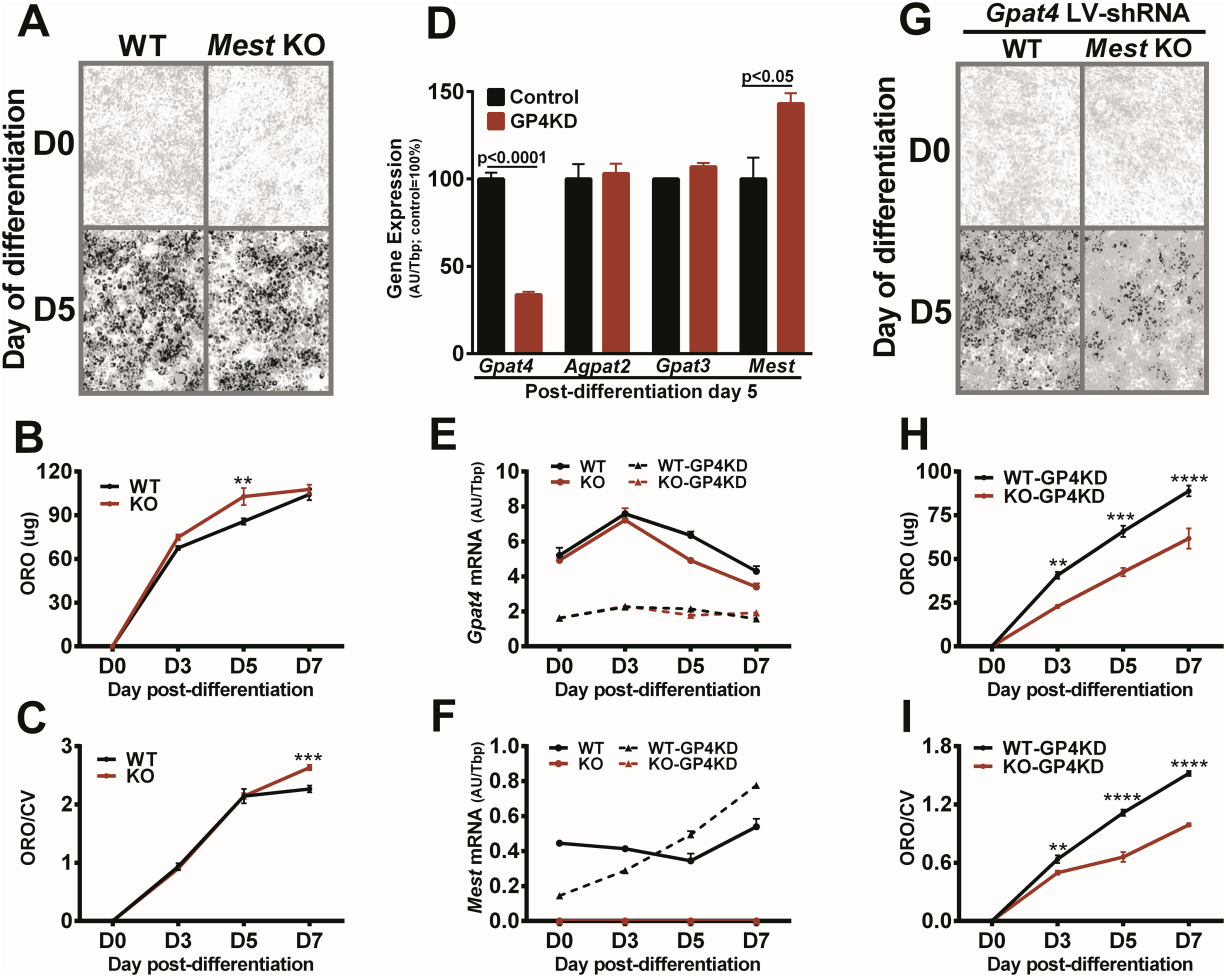
*In vitro* analysis of adipogenesis and effects of *Gpat4* -knockdown on ear-derived mesenchymal progenitor cells from wildtype and *Mest* KO mice. (A) Oil red O (ORO) staining of lipid is shown in representative 10X (gray-scale) microscopic images of ear-derived mesenchymal progenitor cells (EMSC) from WT and homozygous *Mest* knockout (KO) mice prior to (D0) and after 5 days (D5) of adipogenic differentiation. (B) Graph represents spectrophotometric measurements of eluted ORO (ug) during 7 days of adipogenic differentiation using 3–4 replicate cultures of EMSC from WT and *Mest* KO mice for each time point on the X-axis. (C) Data shows an additional independent experiment using 3–4 replicate cultures of EMSC from WT and *Mest* KO mice at each time point during adipogenic differentiation that were stained with ORO and counterstained with crystal violet (CV) to normalize for cell number. (D) Figure shows mRNA expression of *Gpat4*, *Agpat2*, *Gpat3* and *Mest* measured by qRT-PCR in WT EMSC in the absence (Control; n = 3) or presence of lentiviral shRNA knockdown of *Gpat4 (*GP4KD; n = 3) at post-adipogenic differentiation day 5. Gene expression data is presented as arbitrary units normalized to *Tbp* (AU/*Tbp*). Significance in gene expression between control and GP4KD cells was determined using two-tailed unpaired parametric t-tests and p-values between groups are indicated. (E) and (F) show *Gpat4* and *Mest* mRNA expression in duplicate samples for WT and *Mest* KO EMSC in the absence or presence of GP4KD during adipogenic differentiation from D0 to D7. (G) ORO staining of lipid is shown in representative 10X (gray-scale) microscopic images of WT and *Mest* KO EMSC in the presence of GP4KD prior to (D0) and after 5 days (D5) of adipogenic differentiation. (H) Spectrophotometric profiles of eluted ORO during 7 days of adipogenic differentiation are shown for 3–4 replicates of WT and *Mest* KO EMSC after GP4KD are shown for each time point on the X-axis. (I) Data shows an additional independent experiment using 3–4 replicate cultures of EMSC from WT and *Mest* KO mice after GP4KD at each time point during adipogenic differentiation that were stained with ORO and counterstained with CV to normalize for cell number. Significance at each time point of adipogenic differentiation (B, C, H and I) was determined by 2-way ANOVA and Sidak’s multiple comparisons test. Datasets with 2, 3 or 4 asterisks indicate p-values of <0.01, <0.001 and <0.0001 respectively.

*In vivo* inactivation of *Mest* both globally and in adipocytes was shown to reduce adiposity (Figs 2 and 3); therefore, studies were initiated to determine whether similar effects of *Mest* inactivation can be observed in an adipogenic population of primary ear-derived mesenchymal progenitor cells (EMSC) from mice [57]. Results showed that EMSC from *Mest*^**gKO**^ mice were comparable in their capacity for adipogenic differentiation and accumulation of lipids compared to EMSC from WT mice as indicated by the panels showing oil red O (ORO) staining of lipids and crystal violet (CV) staining of cell nuclei (Fig 9A); and, in longitudinal profiles of lipogenesis measured by spectrophotometric determination of ORO in two independent experiments (Fig 9B and 9C). Although these results are somewhat contradictory to what is observed with *in vivo* studies, one possible explanation for the lack of effect of *Mest* inactivation in EMSC adipogenesis could be that cellular access to abundant nutrients and robust expression of lipid biosynthetic glycerol- and acyl-glycerol phosphate acyl transferases (GPATs and AGPATs) simply overwhelms the contribution MEST might have in facilitating lipid accumulation. By using an approach similar to one that used siRNA knockdown of GPAM in HEK293 cells to confirm GPAT-function for AGPAT6 [58], now renamed GPAT4, it was hypothesized that attenuation of GPAT/AGPAT function in EMSC might allow the contribution of MEST in lipid accumulation in EMSC undergoing adipogenesis to emerge. To test this possibility, EMSC were transduced with a lentiviral (LV) construct expressing shRNA against *Gpat4*, a glycerol-3-phosphate O-acyltransferase that catalyzes the acylation of glycerol-3-phosphate in the first and rate limiting step of *de novo* triacylglycerol and glycerophospholipid synthesis. *Gpat4* was selected because, like *Mest*, it is localized within the endoplasmic reticulum (ER) [59] and is expressed during adipogenic differentiation of EMSC derived from both WT and *Mest*^**gKO**^ mice (Fig 9D and 9E). Expression of *Gpat4* in EMSC from WT mice transduced with LV shRNA against *Gpat4* (GP4KD) was reduced ~65–70% 5 days after initiation of adipogenic differentiation (Fig 9D). The expression of *Agpat2* and *Gpat3*, which have 42% and 67% coding sequence identity with *Gpat4* and are highly expressed in EMSC during adipogenic differentiation, are unaffected in the GP4KD EMSC (Fig 9D). Interestingly, *Mest* expression was modestly increased ~30% in GP4KD EMSC which suggests that its activation is a possible compensatory response to the attenuation of GPAT4 function. An independent longitudinal study using EMSC from WT and *Mest*^**gKO**^ mice in the absence or presence of GP4KD shows comparable temporal patterns of *Gpat4* expression during 7 days of adipogenic differentiation among genotypes which was significantly attenuated by the GP4KD (Fig 9E). *Mest* expression in WT GP4KD EMSC was initially lower than WT EMSC during the first 3 days after adipogenic differentiation was initiated (i.e. D0-D3), but was higher than WT EMSC by day 5 post-differentiation (Fig 9F) which is consistent with previous results (Fig 9D). While GP4KD transduced WT and *Mest*^**gKO**^ EMSC showed highly attenuated lipogenesis (Fig 9G) compared to non-transduced cells (Fig 9A), GP4KD was significantly more effective in mitigating lipogenesis in *Mest*^**gKO**^ EMSC compared with WT EMSC as observed in the ORO/CV stained image (Fig 9G) and in the spectrophotometric measurements of ORO stained lipids in two independent longitudinal studies (Fig 9H and 9I). These data suggest that intact MEST function in WT EMSC diminishes GP4KD-mediated reductions of lipogenesis and supports a role for MEST in facilitating lipid accumulation.

## Discussion

Since adipose tissue *Mest* was found to be positively associated with variable development of diet-induced obesity within a genetically homogeneous population of mice [10], a number of studies by our laboratory and others have utilized *Mest* as a sensitive biological indicator for adipocyte hypertrophy and fat expansion [7, 14, 16, 21, 60]. The initial characterization of mice with a targeted inactivation of *Mest* on a mixed genetic background (*Mest*^**tm1Lef**^) did not describe adiposity-related phenotypes; however, these mice were smaller than control animals, had high rates of early post-natal lethality and showed abnormal maternal behavior [43]. Subsequent analyses of a subset of 9-month old *Mest*^**tm1Lef**^ mice on a mixed B6/129 background showed modestly reduced bodyweights and fat pad weights suggesting a role for MEST in the storage of lipid in adipocytes [16]. To avoid problems associated with abnormal maternal behavior and post-natal lethality in *Mest*-deficient mice, which would confound the interpretation of MEST function in diet-induced obesity and adipose tissue expansion (ATE), we generated an isogenic (C57BL/6J) mouse model with *loxP* sites flanking the 3^rd^ exon of *Mest* to selectively inactivate *Mest* in a tissue-specific manner. Surprisingly, the creation of mice with Ella-cre-mediated global inactivation of *Mest* using our targeted model, unlike the *Mest*^**tm1Lef**^ mice [43], did not result in increased risk of early post-natal lethality, reduced post-natal longitudinal growth, or abnormal maternal behavioral phenotypes. The reasons for differences between our targeted *Mest* mouse model and *Mest*^**tm1Lef**^ are not well understood but could be due to differences in the genetic background of the mice, the gene targeting construct, or mutation(s) in the host ES cell used for targeting [61]. Recently it was reported that backcrossing targeted *Mest*^**tm1Lef**^ mice 5 generations onto a129/SVJ background also eliminated the early postnatal lethality and maternal behavioral phenotypes of mice with a paternal inactivation of *Mest*; however, the explanation why this occurred was not clearly defined [62].

A microRNA (*miR-335*) suggested to have a role in the regulation of lipid metabolism, adipogenesis, adipose tissue inflammation and chondrogenesis [47, 48, 63] is located within the 2^nd^ intron of *Mest* TV2 (Fig 1D) and only ~595 bp upstream (5’) from the loxP/NEO cassette used to generate the targeted *Mest* allele. The regulation of miR-335 is complex with some studies suggesting that it is a paternally expressed imprinted microRNA that is co-regulated with *Mest* whereas others suggest that it can be regulated via its own promoter [48, 64]. Since it is plausible that perturbed *miR-335* function could modify ATE in our mouse model, it was necessary to determine whether *miR-335* is dysregulated in mice with targeted *Mest*. Analyses of *miRNA-335-5p* expression in iWAT of 6–7 day old mice, at a time when fat accretion is rapid and *Mest* is highly expressed [16], showed comparable levels of *miR-335* for all genotypes (Fig 1E) with no evidence for dysregulation of *miR-335* in mice with inactivated *Mest*. Normal *miR-335* expression in mice with a targeted allele of *Mest* suggests that phenotypic differences primarily result from loss of MEST function.

HFD-fed mice with inactivation of *Mest* on the paternal allele (*Mest*^**pKO**^) showed significantly reduced bodyweight (Fig 2A), adiposity (Fig 2B and 2C), adipocyte hypertrophy (Fig 4) and improved glucose and insulin tolerance (Fig 4B and 4C); but, relatively few differences in metabolic parameters measured by indirect calorimetry (Fig 4G, 4H, 4I and 4J) compared to WT littermate controls. Although a reduction in bodyweight of just a little over 4 grams between male *Mest*^**pKO**^ and WT mice after being fed a HFD for 8 weeks appears to be modest, this would be equivalent to a 177 vs 200 pound individual with ~75% (~17.3 lbs) of the additional bodyweight being fat mass. Additional studies using *Mest*^**pFL**^ mice with either *Adipoq- or Fabp4*-cre mediated inactivation of the paternal *Mest* allele primarily in adipocytes (Fig 3; S1 Fig) showed similar reductions in both bodyweight and adiposity compared to their respective littermate controls suggesting that inactivation of *Mest* in adipocytes is predominantly responsible for the observed differences in adiposity related phenotypes between genotypes.

A predicted complication in assessing phenotypic differences between WT mice and mice with global or adipose tissue specific inactivation of *Mest* was the expected variability in the development of obesity within the WT cohort and its positive association with WAT MEST [7, 10, 16]. This was in fact the case and was exemplified by the analyses of tertiles (n = 6) of WT mice with the lowest (WT-LM) and highest (WT-HM) eWAT *Mest* mRNA expression (Fig 2D and 2E). Our analyses showed that differences in bodyweight and adiposity were more pronounced when comparing *Mest*^**pKO**^ with WT-HM mice and were consistent with previous studies of interindividual variation of *Mest* in the absence of genetic heterogeneity. When tertiles for male WT mice based on eWAT *Mest* expression are extrapolated to human body weight, WT-HM and WT-LM would weigh 215 vs183 pounds respectively. The lack of difference observed for bodyweight, adiposity, and adipocyte hypertrophy between WT-LM and *Mest*^**pKO**^ mice fed a HFD suggests that ‘basal’ levels of ATE do not require MEST. Additionally, studies showing increased adipocyte hypertrophy in mice with transgenic overexpression of *Mest* [20] coupled with high interindividual variability in WAT *Mest* expression in a genetically homogeneous population of mice [10, 16, 65] supports the premise that MEST could be an ‘epigenetically’ regulated rheostat for the control of ATE beyond defined basal levels established by the genetic background.

A concern with the ablation of *Mest* in a murine mouse model was that it could reduce the capacity for WAT to expand in an obesogenic environment leading to adipose dysfunction, ectopic accumulation of hepatic and intramuscular lipid, and impaired insulin sensitivity similar to that observed in lipodystrophy [66, 67]. However, *Mest*^**pKO**^ mice fed a HFD for 8 weeks showed markedly improved glucose homeostasis compared with WT controls (Fig 4B and 4C) which suggest that the suppression of ATE in *Mest*^**pKO**^ mice does not adversely affect metabolic health of WAT. These observations are supported by reduced WAT expression of markers for inflammation, hypoxia and macrophage infiltration in *Mest*^**pKO**^ and WT-LM mice compared to WT-HM mice (Fig 6) and lack of differences in hepatic expression of genes shown to be associated with the development of hepatic steatosis [49–52]. Data suggests that improved glucose tolerance is at least partially caused by reduced ATE in mice with inactivated or low WAT *Mest* expression. Improved glucose metabolism in association with reduced adipocyte lipid accumulation in mice with deficient *Mest*, caused by reduced adipocyte lipid accumulation shows similarity to the maintenance of normal glucose metabolism by caloric restriction; or, by mechanisms that enhance energy expenditure such as physical activity or cold-activated thermogenesis [68–73].

Although WT mice accrued significantly more bodyweight and adiposity than *Mest*^**pKO**^ mice after being fed a HFD for 8 weeks (Fig 4D and 4E), there were no differences in caloric intake between genotypes when measured during the initial (wk 8) and final week (wk 16) of high fat feeding (Fig 4F). Since indirect calorimetric measurements showed a consistent but non-significant (~4–5%) increase in energy expenditure for *Mest*^**pKO**^ mice during the day and night (Fig 4H) as well as significantly increased nighttime activity (Fig 4I and 4J), it is possible that these small differences in energy expenditure during the 8-week longitudinal study could contribute to some of the phenotypic differences in bodyweight and adiposity between genotypes.

Genes (i.e. *Sfrp5*, *Bmp3*, *Nkd1* and *Serpine1*) expressed in WAT previously identified as being positively correlated with WAT *Mest* mRNA and variable development of adiposity in a genetically homogeneous population of mice fed obesogenic diet [7, 10, 16] were not significantly effected in WAT of *Mest*^**pKO**^ mice (Fig 7B, 7C, 7D and 7E). However, consistent with previous studies, analyses of WAT gene expression in the WT mice used in the HFD study in Fig 2 showed significant positive associations between eWAT *Mest* vs *Sfrp5*, *Bmp3* and *Nkd1*; and, between iWAT *Mest* vs *Sfrp5*, *Bmp3*, *Nkd1* and *Serpine1*. Since these genes were expressed at similar levels in WAT of HFD-fed *Mest*^**pKO**^ and WT-LM mice (Fig 7A, 7B, 7C, 7D and 7E), both of which show comparable weight gain and adiposity (Fig 2E and 2F), this suggests that differences in adiposity rather than WAT MEST affects their expression.

RNA-Seq analyses performed with RNA from eWAT of WT and *Mest*^**pKO**^ mice to gain insight into gene pathways and mechanisms related to MEST function revealed relatively few differences in gene expression (~36 genes) between genotypes when corrected for FDR at a p-value of <0.1 (Fig 8A) which suggests that ablation of *Mest* has relatively modest effects on gene expression in adipose tissue. This is an exciting feature of the association of reduced MEST and ATE with improved glucose/insulin metabolism because, unlike many other anti-obesity therapies, it provides encouragement that targeting the MEST pathway to reduce ATE may have a minimal number of side effects. Pathway analyses of genes with reduced and increased expression in eWAT of *Mest*^**pKO**^ mice (Fig 8A) showed modest associations with immune system-based pathways and metabolism/catabolism of toxins, respectively. The use of a broader set of genes with less stringent criteria for significance (S2 Table) in the gene ontology enrichment analyses also showed genes with reduced expression in eWAT of HFD-fed *Mest*^**pKO**^ mice to be associated with immune system processes (Fig 8B), including inflammation, which is consistent with improved glucose tolerance and insulin sensitivity in these mice. Genes more highly expressed in eWAT of *Mest*^**pKO**^ were categorized within multiple metabolic processes including those associated with metabolism of acyl-CoA, fatty acids and lipids (Fig 8B*)* and included a subset of carboxylesterases (*Ces1d*, *Ces1f*), carnitine palmitoyltransferase 2 (*Cpt2*) and glycerol-3-phosphate acyltransferases (*Gpat4*, *Gpat3;* S2 Table). Since elevated *Mest* is associated with increased adiposity, the increased expression of lipid biosynthetic genes in the absence of MEST may reflect a compensatory response. GPAT4, an integral membrane protein is important for the first step in catalyzing triglyceride synthesis and has been suggested to diffuse from the endoplasmic reticulum (ER) to lipid droplets via membrane bridges [74]. Since evidence suggests that MEST is localized within the ER [16, 20, 27], it’s plausible that interactions between MEST and GPAT4, or with other ER-localized GPATs or AGPATs, could facilitate triglyceride synthesis and storage in adipocytes. Studies are currently underway to delineate temporal patterns of MEST localization within the ER and its proximity to nascent lipid droplet formation.

Catalytic promiscuity and moonlighting are common among α/β-hydrolase fold proteins thus making it difficult to associate a specific catalytic function for MEST based on sequence homology [75–77]. Although the precise catalytic mechanism for MEST has not been defined, evidence showing a role for MEST in augmenting ATE and lipid accumulation in adipocytes [7, 16, 20] coupled with its intracellular localization within the ER [16, 20, 27] and the presence of a catalytic triad associated with proteases, lipases and acyltransferases within its peptide sequence [24, 26], all suggest that MEST acts to facilitate the accumulation of lipid in adipocytes.

The observation that mesenchymal progenitor cells (EMSC) derived from mice with a targeted inactivation of *Mest* (*Mest*^**gKO**^) showed no difference in adipogenic potential and lipid accumulation compared to WT EMSC (Fig 9A, 9B and 9C) is contradictory to results showing increased lipid accumulation in 3T3-L1 adipocytes with transgenic overexpression of *Mest* [20]; and, underscores the complexity involved in understanding MEST function using *in vitro* models. However, in lentiviral shRNA knockdown of GPAT4, a gene whose inactivation in mice causes subdermal lipodystrophy and reduced diet-induced obesity [78], in both WT and *Mest*^**gKO**^ EMSC resulted in the emergence of differences in adipogenesis between the two genotypes with EMSC derived from *Mest*^**gKO**^ mice showing significantly reduced lipid accumulation compared to WT EMSC (Fig 9G, 9H and 9I). These data provide strong evidence that MEST at physiological levels may augment lipid accumulation and storage during adipogenic differentiation by its own endogenous acyltransferase activity, interact with or stabilize proteins associated with fatty acid uptake or lipid droplet formation, or reduce adipogenesis-suppressing intracellular levels of EET [35–37] via endogenous epoxide hydrolase activity. Proteomic and lipodomic studies are needed to further delineate the precise catalytic mechanisms associated with MEST and its role in mediating ATE and glucose homeostasis in WAT.

In summary, our data shows that reduced diet-induced ATE in MEST-deficient mice diminishes expression of markers of hypoxia and inflammation in WAT which leads to improved glucose tolerance and insulin sensitivity. Since minimal additional physiological effects are observed in mice with inactivation of *Mest*, an intervention that represses the function of MEST, or its associated pathway, could be a feasible strategy to mitigate obesity and the inception of T2D.

## Methods

### Gene targeting

A cre/*lox*P system was used to create mice with global and conditional inactivation of mesoderm specific transcript *(Mest)*. DNA amplified from a C57BL/6J genomic BAC clone (RP24-211g11) was used to generate a targeting vector with *lox*P sites flanking exon 3 of *Mest* transcript variant/protein isoform 2 (NM_008590.2). A third *lox*P site flanking a NEO cassette (G418 resistance) 5’ of exon 3 was included as a selectable marker. Targeted C57BL/6J embryonic stem cells carrying the conditional floxed allele were introduced into C57BL/6J-Tyrc-2J host blastocysts and chimeric progeny crossed with C57BL/6J-Tyrc-2J mice to generate mice heterozygous for the targeted allele. The presence and correct orientation of the 3 *lox*P sites with respect to *Mest* exon 3 and the NEO cassette were confirmed via PCR. Generation of mice with global inactivation of *Mest* and with a floxed *Mest* exon 3 in the absence of the NEO cassette was achieved by crossing mice carrying the targeted allele to *Ella*-cre mice which has restricted expression of cre recombinase under the control of the adenoviral *Ella* promoter in oocytes and in preimplantation embryos [79]. Progeny that show positive PCR amplification patterns that correspond to a complete deletion of the floxed region and/or deletion of the NEO cassette were backcrossed to C57BL/6J mice to ensure germline transmission of the mutation and removal of the *Ella*-cre transgene.

### Animals and study design

Since *Mest* is almost exclusively expressed from the paternally-derived allele in adult mice, *e*xperimental cohorts of mice with a paternal inactivation of Mest (*Mest*^**pKO**^) were generated by crossing wildtype (WT) C57BL/6J female mice with male mice heterozygous for the inactivated (KO) *Mest* allele on the maternal allele (*Mest*^**mKO**^). This cross yields equal ratios of WT and *Mest*^**pKO**^ mice. Complete knockout of *Mest* in tissues of *Mest*^**pKO**^ mice have been verified by both gene expression and Western blot analysis (Fig 1B and 1C). Longitudinal dietary obesity studies were performed using male WT and *Mest*^**pKO**^ mice because male C57BL/6J has greater susceptibility for the development of diet-induced obesity than females. Experimental cohorts of mice to study the conditional inactivation of *Mest* were generated via crosses between female mice that are hemizygous for either the *Fabp4*-cre (B6.Cg-Tg (Fabp4-cre)1Rev/J; JAX Stock# 005069) or *Adipoq*-cre (B6;FVB-Tg(Adipoq-cre)1Evdr/J; JAX Stock# 010803) transgenes with male mice containing either a maternal (*Mest*^**mFL**^) or paternal (*Mest*^**pFL**^) floxed allele for *Mest*. The resulting genotypes will include mice with *Fabp4*- or Adipoq-cre inactivation of the paternal allele for *Mest* (*Mest* ^**FpKO**^ or *Mest* ^**ApKO**^ respectively) and control groups consisting of WT, WT-cre (*Fabp4*- or *Adipoq*-cre) and *Mest*^**pFL**^ mice. Prior to use in generating experimental mice, the *Adipoq*-cre transgenic mice were crossed to C57BL/6J for several subsequent generations to eliminate potential genomic contribution from the FVB strain. Genomic analyses of the Adipoq-cre using a 1449 SNP marker panel (Taconic, Germantown, NY) confirms 99.79% C57BL/6J with the only residual genome of FVB located between ~83.5 and ~92.1 mb on mouse *Chr 9*, the putative genomic insertion site of the *Adipoq*-cre transgene [80].

The animals were maintained in a temperature-controlled room (23°C) with a 12-h light/12-hr dark cycle. For experiments performed at Pennington Biomedical Research Center, the mice were reared under conventional conditions and fed PicoLab Rodent Diet 20 (Lab Diet; 13 kcal% fat) after weaning. For experiments conducted at Maine Medical Center Research Institute Animal Facility, the mice were kept in a barrier facility and given 2018 Teklad Global 18% Protein Rodent Diet (Harlan; 18 kcal% fat) post-weaning. For dietary obesity studies mice were singly-housed at 7 weeks of age and then fed a high fat diet (HFD; D12331, Research Diets; 58 kcal% fat; 23.26 kJ/g) starting a 8 weeks of age for 8–16 weeks as indicated for each study cohort. The design used for the dietary obesity studies for each of the cohorts of mice are shown in S3 Table. Upon completion of the studies, all mice were sacrificed by anesthesia with isoflurane followed by cervical dislocation according to accepted guidelines and tissues were harvested for analyses. All animal experiments and methods were approved by the Institutional Animal Care and Use Committee at The Pennington Biomedical Research Center (Baton Rouge, LA) and The Maine Medical Center Research Institute (Scarborough, ME) in accordance with National Institutes of Health guidelines for care and use of laboratory animals.

### Glucose and insulin tolerance tests

Glucose tolerance test (GTT) was performed in 7 week old male mice prior to initiation of high fat diet and repeated after 8 weeks on high fat diet. Mice were fasted overnight and administered 2 g/kg BW glucose intraperitoneally (IP). Blood glucose levels were determined using Accu-check Aviva plus system (Roche Diagnostics). Insulin tolerance test was conducted in mice fed high fat diet for 8 weeks. Mice were fasted for 6 hours and injected with human recombinant insulin (Humulin-R, Eli Lilly and Company) at a dose of 1.5 kg/BW IP.

### Indirect calorimetry

The Promethion Metabolic Cage System (Sable Systems) was used for indirect calorimetry. The mice were acclimated to the system for 12-hrs followed by 72-hr data collection.

### Determination of body composition

#### Nuclear magnetic resonance

Body composition (body fat, lean mass and free fluid) was analyzed by Minispec NMR (Bruker) which allowed for non-invasive, serial body composition measurements in the mouse. The Minispec uses the contrasting hydrogen density and/or hydrogen spin properties from adipose tissue and muscle for estimating body composition. A quality control check of NMR parameters using a standard provided by the manufacturer was performed at the beginning of each day of testing.

#### Dual-energy X-ray absorptiometry

Mice were scanned using the Lunar PIXImus densitometer (GE Medical Lunar; DEXA). Whole-body and femoral areal bone mineral density, and body composition excluding the head were determined in each mouse under isoflurane anesthesia (IsoFlo, Abbot Labs). The Piximus was calibrated at the beginning of each day of testing using a phantom according to the manufacturer’s instructions.

### Histology and morphometrics of white adipose tissue

Inguinal (iWAT) and epididymal (eWAT) adipose tissue were fixed in Bouin’s solution (Sigma-Aldrich), and paraffin-embedded sections were stained with H & E. Adipocyte area and size distribution were analyzed using Image J software at specific parameters to measure area of each adipocyte based on size and exclusion limits.

### Mest transcript variants

RNA was extracted from eWAT and iWAT of wild-type mice (Mest^+/+^) homogenized in TriReagent (Molecular Research Center, Inc.) and then purified using RNeasy Mini Kit and RNase-free DNAse (Qiagen). Isolated RNA was protected from RNAse contamination with SUPERase-In (Life Technologies). RNA quantity and quality was determined using Nanodrop 1000 spectrophotometer. Specific primers against the *Mest* transcript variants 1(TV1; forward: 5’-CCCTGTGATCCGCAATCCT-3’; reverse: 5’-ACTACTGTCTGCATTTGGGCTATG-3’), 2 (TV2; forward: 5’-GCGGCATGGGATAATGC-3’; reverse: 5’-CTACTTGGACCCACCACTCTCT-3’) and 3 (TV3; forward: 5’-GGGTAGAGAGAAAAAGTGTGGAA-3’; reverse: 5’-CCTCTAAGGAACAGCGACTTC-3’) were designed using Primer Express software v3.0.1 (Life Technologies) to yield amplicons ranging from 72 to 99 bp. White adipose tissue cDNA was synthesized using High-Capacity cDNA Reverse Transcription Kit (Life Technologies). Quantitative PCR was performed using 20 ng cDNA input and yield was compared between 25, 30 and 36 PCR cycles. PCR products were resolved using 3% GenePure LE agarose (ISC Bioexpress) in 1X TBE running buffer at 80 volts for 20 mins. Gel image was obtained using GelDoc-ItTS2 Imaging System (UVP).

### Quantitative reverse transcription PCR

#### Expression of mRNA

Quantitative reverse transcription-PCR was performed using total RNA with specific primers and probes (S3 Table) designed using Primer Express software v3.0.1 (Life Technologies) essentially as previously described [16] except that a BioRad CFX Real-Time System was also used for analyses. TaqMan probes (Biosearch Technologies) were used for gene quantification using TaqMan^®^ RNA-to-CT 1-Step Kit (Life Technologies). Gene expression data were normalized to level of cyclophilin b (*Ppib*), or TATA box binding protein (*Tbp*). In addition, the total nanograms of input RNA/reaction was quantified using Qubit HS RNA Assay Kit (Life Technologies) and can be used to normalize gene expression by RNA input if necessary.

#### Expression of miRNA

Total RNA including miRNAs was isolated from white adipose tissue using miRNeasy mini kit (Qiagen). cDNA synthesis was performed using the TaqMan^®^ MicroRNA Reverse Transcription Kit and TaqMan miRNA specific primers (Life Technologies) for mmu-miR-335-5p, mmu-miR-335-3p and snoRNA202 (NCBI Accession #AF357327). The cDNA was amplified using the TaqMan Universal Mastermix II, no UNG and TaqMan Small RNA Assay (Life Technologies). Quantitive PCR was performed using the CFX384 Real Time PCR detection system (BioRad). Relative abundance of miR-335 was quantified using the 2^-ΔΔCT^ method using snoRNA202 for normalization of expression.

### Western blot analyses

Total tissue lysate from eWAT and iWAT were prepared in RIPA buffer containing 1% protease, phosphatase I and phosphatase II inhibitors (Sigma). Protein content was determined using Sigma BCA protein assay kit. Proteins were resolved on 10% SDS-PAGE gel and after separation electro-transferred to PVDF (Immobilon-P, EMD Millipore) or nitrocellulose (Hybond-ECL, GE Healthcare) membranes. Blots were incubated in antibodies against MEST (1:5000) and GAPDH (1:1000, ABCAM ab9484). Bands were visualized and quantified using the Odyssey imaging system (Li-Cor Bioscience).

### *In vitro* cell culture

Experimental cohorts for *in vitro* experiments were generated by intercrossing *Mest* heterozygous mice to generate WT (+/+), *Mest*^**pKO**^ (+/KO) and *Mest*^**gKO**^ (KO/KO) mice. Ear mesenchymal stem cells (EMSCs) were isolated from WT and *Mest*^**gKO**^ mice as previously described [57]. Cells were cultured in 5% CO_2_ and maintained in DMEM/F-12 (Life Technologies) supplemented with 10% FBS (Life Technologies) supplemented with Primocin as an antibiotic (InVivoGen), and 10 ng/ml recombinant bFGF (PeproTech). To induce adipocyte differentiation, recombinant bFGF was removed and replaced with 10% FBS with 0.5 mM methylisobutylxanthine, 1 uM dexamethasone, 5 ug/ml insulin, and 5 uM troglitazone. On day 2, cells were fed 5 ug/ml insulin plus 5 uM troglitazone. On day 4 and every 2 days thereafter, cells were fed with 15% FBS. Lipid droplets were visualized using Oil Red O staining. Cells were washed with PBS followed by fixation with 10% neutral buffered formalin for 10 min. The cells were then washed once in dH_2_O and incubated in 60% isopropanol for 5 min. The isopropanol was removed and followed by an incubation in Oil Red O staining solution (0.35% Oil Red O in 60% isopropanol) for 10 min, washed once in 60% isopropanol and 3X for 10 min with dH_2_O. Oil Red O dye was eluted in 100% isopropanol for 15 minutes. The degree of adipogenic differentiation was quantified spectrophotometrically by measuring absorbance at 490 nm. A standard curve with known amounts of Oil Red O dye (ug/ml) was used to quantify lipid as total ug of Oil Red O eluted. After elution of Oil Red O dye, cells were then washed 1X with 100% isopropanol followed by dH_2_O and subsequently used for quantifying relative cell number by nuclear staining with crystal violet. Relative cell number was measured by staining cells with 0.05% crystal violet in deionized H_2_O for 5 minutes. The cells were then washed 3X for 10 min with dH_2_O then lysed in 1% SDS. Crystal violet in the 1% SDS lysate was measured at an absorbance of 560 nm a standard curve with known amounts of crystal violet (ug/ml) was used to quantify relative cell number and to normalize Oil Red O eluted from cells.

### Gene knockdown

Five short-hairpin RNA (shRNA) constructs against *Gpat4* (Agpat6) in lentiviral vector pLKO.1 were obtained from Sigma-Aldrich. For knockdown experiments, the construct chosen was TRCN0000099245. According to the manufacturer’s protocol, EMSC were transduced in maintenance medium in the presence of polybrene (Sigma-Aldrich). Transduction was performed at MOI = 2. After overnight incubation, virus-containing media was removed and standard culture media was added. Cells were allowed to expand for 48 hours and then selection was initiated using puromycin (Sigma-Aldrich) at 4 ug/ml. Puromycin-resistant cells were expanded until P6 and then seeded in 12-well plates at a density of 100,000 cells per well. Prior to lentiviral transduction, a titration was performed to determine the minimum concentration of puromycin required to cause complete cell death after 3–5 days. Cells were harvested for gene expression, Oil Red O and crystal violet staining.

### SOLiD SAGE analyses

SAGE gene expression profiling was performed with next-generation sequencing using the Life Technologies 5500XL SOLiD system. RNA was isolated from tissues using TRI Reagent; quantified and quality was determined using an Agilent 2100 Bioanalyzer. RNA was transcribed into cDNA via priming at the poly A+ tail. Double-stranded cDNA was digested with *Nla*III restriction enzyme and ligated to adaptors containing an EcoP15L restriction site. Digestion was performed with *EcoP*15L and ligation of "bar code" oligonucleotides that specifically label each sample, and subsequent PCR amplification yielded a library for sequencing where each gene was represented with a 3'-expression tag. The alignment of sequence tags back to a reference genome was performed using a modified version of the Applied Biosystems SOLiD^™^ SAGE^™^ Analysis Software v1.10 that was installed, modified to meet stringent mapping criteria, and extensively tested in-house. RNA isolated from eWAT of 6 independent wildtype control mice and 5 *Mest*^**pKO**^ mice were used for preparation of libraries and sequencing analyses. Raw count files were analyzed by the R/Bioconductor program DE-Seq31; significance and predictive analyses were performed using SAM (Significance Analysis of Microarrays; http://www-stat.stanford.edu/~tibs/SAM/index.html) and PAM (Prediction Analysis for Microarrays; http://statweb.stanford.edu/~tibs/PAM/index.html) respectively [81, 82]. P-values were adjusted for false discovery rate using the Benjamini-Hochberg method [83]. Pairwise analysis and hierarchical clustering of samples was performed using Bioconductor edgeR Version 3.2.3 and JMP Genomics respectively. Raw sequencing files from ABI SOLiD SAGE analyses have been deposited in NCBI’s Gene Expression Omnibus (GEO; https://www.ncbi.nlm.nih.gov/geo/) with the accession number GSE96968.

### Statistics

Statistical calculations were performed using GraphPad Prism software V.6 and Microsoft Excel 2010. Differences between 2 groups were calculated using two-tailed unpaired parametric t-test with confidence level at 95%. Significance among multiple groups was calculated with ordinary one-way ANOVA followed by Tukey’s multiple comparisons test with an alpha of 0.5 or by the Fisher’s LSD test as indicated. Significance at each time point of longitudinal phenotypic datasets was determined using 2-way ANOVA with Holm-Sidak multiple comparisons test. Data is presented as the mean ± SEM. P<0.05 was considered to be significant.

## Ethics statement

The use of murine models for the studies presented in the submitted manuscript was approved by the Institutional Animal Care and Use Committees (IACUC) at the Pennington Biomedical Research Center and the Maine Medical Center Research Institutes.

## Supporting information

S1 Fig Dietary obesity in mice with *Fabp4*- cre mediated inactivation of *Mest*. (A) *Mest* mRNA expression in inguinal (iWAT) and epididymal (eWAT) white adipose tissue of wildtype (WT; n = 8), WT.*Fabp4*-cre (WT-cre; n = 11), paternal floxed *Mest* (pFL; n = 6) and pFL.*Fabp4*-cre (FpKO; n = 12) mice after being fed a high fat diet (HFD) from 8 to 20 weeks of age. *Mest* mRNA expression measured by TaqMan QRT-PCR is represented as the mean ± SEM arbitrary units (AU) normalized to cyclophilin b (*Ppib*). Significance in *Mest* RNA expression between groups was determined via one-way ANOVA using Tukey’s multiple comparisons test. (B) Data shows the longitudinal measurements of bodyweight (BWT) for WT (n = 8), WT-cre (n = 11), pFL (n = 6) and FpKO (n = 12) mice fed a HFD from 8 to 20 weeks of age as indicated by the arrow along the X-axis. (C) Longitudinal measurements of adiposity index; (D) fat-free mass (g) and (E) fat mass (g) measured by NMR at the times indicated on the X-axis are shown for WT (n = 8), WT-cre (n = 11), pFL (n = 6) and FpKO (n = 12) mice fed a HFD from 8 to 20 weeks of age as indicated by the arrow along the X-axis. (B-E) All data in the longitudinal studies are presented as the mean ± SEM. Significance at each time point of the longitudinal phenotypic analyses was determined by 2-way ANOVA and Tukey’s multiple comparisons test. Time points annotated with ‘a’, ‘b’, ‘c’, and ‘d’ indicate significant differences between ‘FpKO vs pFL’; ‘FpKO vs WT’; ‘FpKO vs WT-cre’ and ‘FpKO vs all genotypes respectively. (F) Data shows morphometric analyses of adipocyte size in eWAT in WT (n = 8), WT-cre (n = 11), pFL (n = 6) and FpKO (n = 12) mice. Two-tailed unpaired parametric t-tests were used to determine significant differences in adipocyte size (F) between genotypes. Datasets annotated with the same letter indicate no significant differences between groups.
(TIF)

S1 Table Differential gene expression in eWAT: WT vs pKO; FDR padj ≤ 0.1. (DOCX)

S2 Table Differential gene expression in eWAT; WT vs pKO; pvalue ≤ 0.05. (DOCX)

S3 Table Murine cohorts and study design. (DOCX)

S4 Table Primer and probe sets: qRT-PCR. (DOCX)
