# Supplementary figures and images for: Diet-induced adipose tissue expansion is mitigated in mice with a targeted inactivation of mesoderm specific transcript (Mest)
Source: PLoS One. 2017 Jun 22;12(6):e0179879. doi: 10.1371/journal.pone.0179879 (PMC5481029; doi:10.1371/journal.pone.0179879)

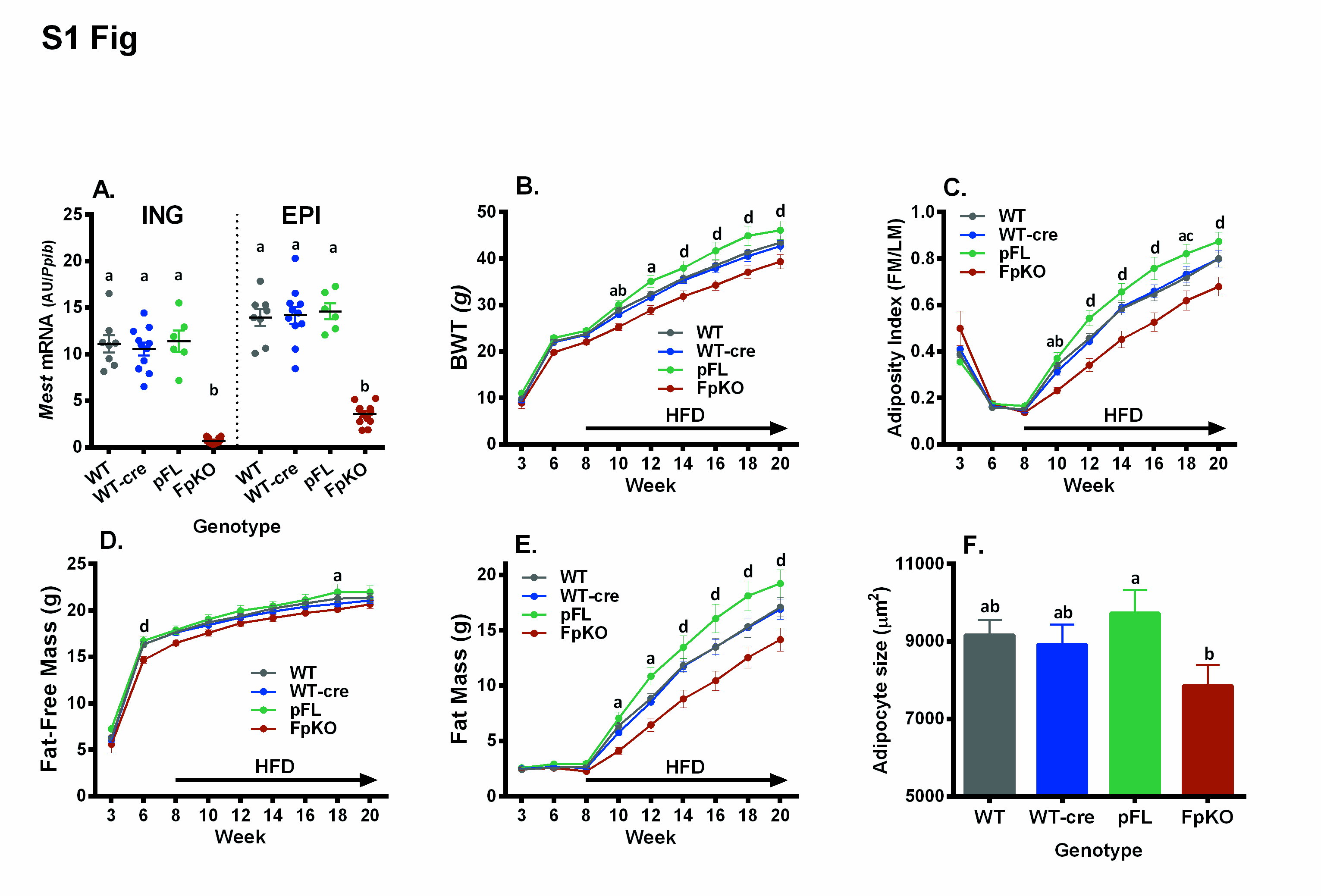

Supplement: S1 Fig — (A) Mest mRNA expression in inguinal (iWAT) and epididymal (eWAT) white adipose tissue of wildtype (WT; n = 8), WT.Fabp4-cre (WT-cre; n = 11), paternal floxed Mest (pFL; n = 6) and pFL.Fabp4-cre (FpKO; n = 12) mice after being fed a high fat diet (HFD) from 8 to 20 weeks of age. Mest mRNA expression measured by TaqMan QRT-PCR is represented as the mean ± SEM arbitrary units (AU) normalized to cyclophilin b (Ppib). Significance in Mest RNA expression between groups was determined via one-way ANOVA using Tukey’s multiple comparisons test. (B) Data shows the longitudinal measurements of bodyweight (BWT) for WT (n = 8), WT-cre (n = 11), pFL (n = 6) and FpKO (n = 12) mice fed a HFD from 8 to 20 weeks of age as indicated by the arrow along the X-axis. (C) Longitudinal measurements of adiposity index; (D) fat-free mass (g) and (E) fat mass (g) measured by NMR at the times indicated on the X-axis are shown for WT (n = 8), WT-cre (n = 11), pFL (n = 6) and FpKO (n = 12) mice fed a HFD from 8 to 20 weeks of age as indicated by the arrow along the X-axis. (B-E) All data in the longitudinal studies are presented as the mean ± SEM. Significance at each time point of the longitudinal phenotypic analyses was determined by 2-way ANOVA and Tukey’s multiple comparisons test. Time points annotated with ‘a’, ‘b’, ‘c’, and ‘d’ indicate significant differences between ‘FpKO vs pFL’; ‘FpKO vs WT’; ‘FpKO vs WT-cre’ and ‘FpKO vs all genotypes respectively. (F) Data shows morphometric analyses of adipocyte size in eWAT in WT (n = 8), WT-cre (n = 11), pFL (n = 6) and FpKO (n = 12) mice. Two-tailed unpaired parametric t-tests were used to determine significant differences in adipocyte size (F) between genotypes. Datasets annotated with the same letter indicate no significant differences between groups. (TIF) [file pone.0179879.s001.tif]
