# Supplementary material for: Diet-induced adipose tissue expansion is mitigated in mice with a targeted inactivation of mesoderm specific transcript (Mest)
Source: PLoS One. 2017 Jun 22;12(6):e0179879. doi: 10.1371/journal.pone.0179879 (PMC5481029; doi:10.1371/journal.pone.0179879)
Supplement: S1 Table — (DOCX) [file pone.0179879.s002.docx]

| **S1 Table. Differential Gene Expression in eWAT: WT vs pKO; FDR padj ≤ 0.1.** | | | | | |
| --- | --- | --- | --- | --- | --- |
| **Gene ID** | **Mean pKO (n=5)** | **Mean WT (n=6)** | **FC (WT/pKO)** | **pval^a^** | **padj (FDR)^b^** |
| ***Chpf*** | **398** | **152** | **0.38** | **1.2E-06** | **0.0018** |
| ***Krt14*** | **61** | **23** | **0.38** | **0.00021** | **0.094** |
| ***Tst*** | **137** | **57** | **0.42** | **4.2E-07** | **0.00072** |
| ***Cyp2e1*** | **1985** | **837** | **0.42** | **2.6E-05** | **0.020** |
| ***S100a1*** | **2813** | **1268** | **0.45** | **3.6E-06** | **0.0051** |
| ***Ces1f*** | **998** | **460** | **0.46** | **8.0E-05** | **0.053** |
| ***Gsta3*** | **10006** | **5206** | **0.52** | **5.4E-06** | **0.0070** |
| ***Plagl1*** | **695** | **381** | **0.55** | **2.0E-05** | **0.016** |
| ***Negr1*** | **715** | **418** | **0.58** | **0.00012** | **0.062** |
| ***Ucp2*** | **1167** | **1953** | **1.67** | **8.9E-05** | **0.053** |
| ***Atp6v0a1*** | **657** | **1101** | **1.68** | **0.00013** | **0.066** |
| ***Mrap*** | **716** | **1225** | **1.71** | **0.00011** | **0.062** |
| ***Dpep2*** | **135** | **243** | **1.80** | **7.1E-05** | **0.050** |
| ***Lbp*** | **6624** | **12404** | **1.87** | **7.2E-06** | **0.0076** |
| ***Adssl1*** | **325** | **622** | **1.91** | **0.00024** | **0.10** |
| ***Mrc2*** | **169** | **326** | **1.93** | **1.2E-05** | **0.011** |
| ***Lilrb3*** | **69** | **135** | **1.96** | **8.9E-05** | **0.053** |
| ***Irf8*** | **131** | **262** | **2.00** | **1.2E-05** | **0.011** |
| ***Gla*** | **94** | **191** | **2.04** | **8.6E-06** | **0.0083** |
| ***Trappc6a*** | **95** | **195** | **2.06** | **6.5E-06** | **0.0076** |
| ***Otop1*** | **60** | **127** | **2.11** | **6.1E-05** | **0.045** |
| ***Lipf*** | **100** | **216** | **2.16** | **7.3E-06** | **0.0076** |
| ***Hp*** | **47204** | **102374** | **2.17** | **2.9E-08** | **0.000065** |
| ***Slc5a7*** | **852** | **1885** | **2.21** | **1.4E-08** | **0.000044** |
| ***AF251705*** | **107** | **240** | **2.25** | **0.00021** | **0.094** |
| ***Lrg1*** | **4048** | **9143** | **2.26** | **9.6E-09** | **0.000044** |
| ***Ccdc109b*** | **122** | **286** | **2.34** | **3.0E-08** | **0.000065** |
| ***Pop4*** | **53** | **131** | **2.47** | **0.00019** | **0.091** |
| ***Ubd*** | **1174** | **2914** | **2.48** | **8.7E-05** | **0.053** |
| ***Atp6v0d2*** | **108** | **290** | **2.69** | **1.2E-08** | **0.000044** |
| ***Tfr2*** | **50** | **135** | **2.71** | **1.2E-08** | **0.000044** |
| ***Mstn*** | **14** | **41** | **3.01** | **0.00012** | **0.062** |
| ***Trdn*** | **30** | **111** | **3.65** | **3.4E-08** | **0.000065** |
| ***Tph2*** | **120** | **540** | **4.52** | **0.00021** | **0.094** |
| ***Mest*** | **1663** | **25107** | **15.09** | **1.6E-65** | **2.5E-61** |
| ***9030619P08Rik*** | **128** | **261** | **2.03** | **0.00022** | **0.096** |

eWAT, epididymal white adipose tissue; WT, wildtype mice; pKO, *Mest***^pKO^** mice; FC, fold change.

^a^p-values were calculated using SAM as described in Materials and Methods.

^b^p-values were adjusted for false discovery rate using the Benjamini-Hochberg method.
