# Supplementary material for: Diet-induced adipose tissue expansion is mitigated in mice with a targeted inactivation of mesoderm specific transcript (Mest)
Source: PLoS One. 2017 Jun 22;12(6):e0179879. doi: 10.1371/journal.pone.0179879 (PMC5481029; doi:10.1371/journal.pone.0179879)
Supplement: S2 Table — (DOCX) [file pone.0179879.s003.docx]

| **S2 Table. Differential Gene Expression in eWAT; WT vs pKO; pvalue ≤ 0.05.** | | | | | |
| --- | --- | --- | --- | --- | --- |
| **Gene ID** | **Mean pKO (n=5)** | **Mean WT (n=6)** | **FC (WT/pKO)** | **pval^a^** | **padj (FDR)^b^** |
| ***Mrgprg*** | **110** | **15** | **0.14** | **0.038** | **0.85** |
| ***Alb*** | **941** | **310** | **0.33** | **0.00029** | **0.11** |
| ***Chpf*** | **398** | **152** | **0.38** | **1.2E-06** | **0.0018** |
| ***Krt14*** | **61** | **23** | **0.38** | **0.00021** | **0.094** |
| ***Coasy*** | **214** | **83** | **0.39** | **0.0089** | **0.54** |
| ***Rbp4*** | **4725** | **1915** | **0.41** | **0.0028** | **0.37** |
| ***Tst*** | **137** | **57** | **0.42** | **4.2E-07** | **0.00072** |
| ***0610007P14Rik*** | **272** | **114** | **0.42** | **0.031** | **0.82** |
| ***Cyp2e1*** | **1985** | **837** | **0.42** | **2.6E-05** | **0.020** |
| ***Ly6e*** | **6024** | **2694** | **0.45** | **0.017** | **0.67** |
| ***S100a1*** | **2813** | **1268** | **0.45** | **3.6E-06** | **0.0051** |
| ***Ces1d*** | **9466** | **4327** | **0.46** | **0.0013** | **0.24** |
| ***Ces1f*** | **998** | **460** | **0.46** | **8.0E-05** | **0.053** |
| ***Glis3*** | **55** | **26** | **0.46** | **0.0041** | **0.40** |
| ***Slc4a10*** | **52** | **25** | **0.49** | **0.011** | **0.56** |
| ***Smpd3*** | **131** | **65** | **0.50** | **0.016** | **0.65** |
| ***Krt7*** | **444** | **225** | **0.51** | **0.043** | **0.88** |
| ***Pck1*** | **27356** | **14044** | **0.51** | **0.0086** | **0.53** |
| ***Gm19426*** | **80** | **41** | **0.51** | **0.013** | **0.59** |
| ***Prrt4*** | **51** | **26** | **0.51** | **0.0036** | **0.38** |
| ***Ephx2*** | **472** | **244** | **0.52** | **0.0045** | **0.40** |
| ***Kctd13*** | **104** | **54** | **0.52** | **0.0044** | **0.40** |
| ***Gsta3*** | **10006** | **5206** | **0.52** | **5.4E-06** | **0.0070** |
| ***Tfrc*** | **1122** | **584** | **0.52** | **0.0012** | **0.22** |
| ***Meox2*** | **977** | **510** | **0.52** | **0.0037** | **0.38** |
| ***Ly6d*** | **133** | **70** | **0.52** | **0.025** | **0.77** |
| ***Unc119*** | **457** | **242** | **0.53** | **0.0035** | **0.38** |
| ***2310061I04Rik*** | **134** | **71** | **0.53** | **0.020** | **0.72** |
| ***Prosc*** | **633** | **338** | **0.53** | **0.0020** | **0.29** |
| ***Cyc1*** | **1877** | **1017** | **0.54** | **0.0024** | **0.34** |
| ***Prpsap2*** | **576** | **313** | **0.54** | **0.0019** | **0.29** |
| ***Mrpl42*** | **706** | **386** | **0.55** | **0.0011** | **0.22** |
| ***Plagl1*** | **695** | **381** | **0.55** | **2.0E-05** | **0.016** |
| ***Smarcd1*** | **140** | **77** | **0.55** | **0.021** | **0.73** |
| ***Fam180a*** | **66** | **37** | **0.55** | **0.010** | **0.56** |
| ***Mrps22*** | **385** | **214** | **0.56** | **0.011** | **0.57** |
| ***Cyp2s1*** | **76** | **43** | **0.56** | **0.0026** | **0.35** |
| ***Hectd2*** | **99** | **56** | **0.57** | **0.030** | **0.81** |
| ***Orm3*** | **427** | **243** | **0.57** | **0.034** | **0.84** |
| ***Zfp560*** | **142** | **81** | **0.57** | **0.00069** | **0.16** |
| ***Gpx5*** | **62** | **36** | **0.58** | **0.021** | **0.73** |
| ***Amotl2*** | **4577** | **2647** | **0.58** | **0.035** | **0.84** |
| ***Fam19a5*** | **100** | **59** | **0.58** | **0.0026** | **0.35** |
| ***Negr1*** | **715** | **418** | **0.58** | **0.00012** | **0.062** |
| ***Ppox*** | **77** | **45** | **0.59** | **0.0038** | **0.38** |
| ***Tfpi*** | **201** | **118** | **0.59** | **0.012** | **0.58** |
| ***Agk*** | **326** | **191** | **0.59** | **0.0060** | **0.45** |
| ***Klb*** | **267** | **157** | **0.59** | **0.00053** | **0.14** |
| ***Tcea3*** | **149** | **88** | **0.59** | **0.0014** | **0.25** |
| ***Hoxb5*** | **125** | **74** | **0.59** | **0.0028** | **0.37** |
| ***Hba-a2*** | **439** | **260** | **0.59** | **0.032** | **0.83** |
| ***Hoxb2*** | **60** | **36** | **0.60** | **0.0099** | **0.55** |
| ***Bnc1*** | **98** | **59** | **0.60** | **0.0024** | **0.34** |
| ***2700046G09Rik*** | **51** | **30** | **0.60** | **0.019** | **0.70** |
| ***Itgb4*** | **209** | **125** | **0.60** | **0.0080** | **0.51** |
| ***Aif1l*** | **168** | **100** | **0.60** | **0.048** | **0.89** |
| ***AI661453*** | **51** | **30** | **0.60** | **0.041** | **0.86** |
| ***Adrb3*** | **4229** | **2530** | **0.60** | **0.0032** | **0.38** |
| ***Eci3*** | **197** | **118** | **0.60** | **0.014** | **0.61** |
| ***Lace1*** | **115** | **69** | **0.60** | **0.031** | **0.82** |
| ***Nap1l3*** | **304** | **183** | **0.60** | **0.0064** | **0.45** |
| ***Nudt8*** | **447** | **269** | **0.60** | **0.023** | **0.74** |
| ***Patz1*** | **149** | **90** | **0.60** | **0.035** | **0.84** |
| ***Sfrp2*** | **442** | **268** | **0.61** | **0.0063** | **0.45** |
| ***Hba-a1*** | **525** | **318** | **0.61** | **0.030** | **0.81** |
| ***Dnm1*** | **658** | **399** | **0.61** | **0.0043** | **0.40** |
| ***LOC101055764*** | **190** | **115** | **0.61** | **0.0098** | **0.55** |
| ***Pgcp*** | **625** | **380** | **0.61** | **0.013** | **0.59** |
| ***Pigl*** | **69** | **42** | **0.61** | **0.0098** | **0.55** |
| ***Fam46b*** | **95** | **58** | **0.61** | **0.0048** | **0.40** |
| ***Fbxw8*** | **2248** | **1375** | **0.61** | **0.028** | **0.78** |
| ***Dcxr*** | **1183** | **724** | **0.61** | **0.038** | **0.85** |
| ***Upk1b*** | **816** | **499** | **0.61** | **0.00039** | **0.12** |
| ***Krt19*** | **610** | **374** | **0.61** | **0.00055** | **0.14** |
| ***Mtfp1*** | **232** | **142** | **0.61** | **0.0015** | **0.26** |
| ***Trim25*** | **561** | **344** | **0.61** | **0.045** | **0.88** |
| ***Btbd2*** | **288** | **177** | **0.61** | **0.020** | **0.72** |
| ***Echdc3*** | **759** | **468** | **0.62** | **0.00075** | **0.17** |
| ***Gm98*** | **115** | **71** | **0.62** | **0.0038** | **0.38** |
| ***Men1*** | **131** | **81** | **0.62** | **0.0063** | **0.45** |
| ***Thap6*** | **85** | **53** | **0.62** | **0.012** | **0.59** |
| ***Acbd3*** | **337** | **209** | **0.62** | **0.044** | **0.88** |
| ***Atp10d*** | **87** | **54** | **0.62** | **0.010** | **0.56** |
| ***Egr1*** | **130** | **81** | **0.62** | **0.029** | **0.79** |
| ***8430423G03Rik*** | **1659** | **1034** | **0.62** | **0.0014** | **0.25** |
| ***Cxadr*** | **295** | **184** | **0.62** | **0.024** | **0.75** |
| ***Met*** | **840** | **525** | **0.63** | **0.021** | **0.73** |
| ***Msln*** | **184** | **115** | **0.63** | **0.050** | **0.90** |
| ***Gpd2*** | **400** | **251** | **0.63** | **0.0016** | **0.26** |
| ***Slc4a4*** | **95** | **60** | **0.63** | **0.0060** | **0.45** |
| ***Sqle*** | **73** | **46** | **0.63** | **0.046** | **0.89** |
| ***Pdhb*** | **1050** | **659** | **0.63** | **0.0096** | **0.55** |
| ***Pcca*** | **1046** | **658** | **0.63** | **0.032** | **0.83** |
| ***Dnase1l3*** | **107** | **67** | **0.63** | **0.033** | **0.83** |
| ***Fam13a*** | **163** | **103** | **0.63** | **0.0034** | **0.38** |
| ***Rad9*** | **435** | **274** | **0.63** | **0.0076** | **0.51** |
| ***Agpat9*** | **1857** | **1172** | **0.63** | **0.043** | **0.88** |
| ***Nhsl1*** | **111** | **70** | **0.63** | **0.012** | **0.58** |
| ***Fndc1*** | **600** | **380** | **0.63** | **0.0016** | **0.27** |
| ***Car3*** | **60367** | **38466** | **0.64** | **0.018** | **0.68** |
| ***Nhedc2*** | **167** | **107** | **0.64** | **0.0034** | **0.38** |
| ***Bcat2*** | **808** | **515** | **0.64** | **0.015** | **0.63** |
| ***1700012D01Rik*** | **58** | **37** | **0.64** | **0.024** | **0.76** |
| ***Homer2*** | **87** | **55** | **0.64** | **0.015** | **0.63** |
| ***B430010I23Rik*** | **77** | **49** | **0.64** | **0.014** | **0.61** |
| ***Skiv2l*** | **106** | **68** | **0.64** | **0.049** | **0.90** |
| ***Enox1*** | **83** | **54** | **0.64** | **0.015** | **0.63** |
| ***Cobl*** | **50** | **32** | **0.64** | **0.035** | **0.84** |
| ***Dtna*** | **128** | **83** | **0.64** | **0.014** | **0.61** |
| ***Acot2*** | **477** | **308** | **0.65** | **0.0022** | **0.32** |
| ***Rad54l*** | **69** | **45** | **0.65** | **0.050** | **0.90** |
| ***Epas1*** | **7589** | **4923** | **0.65** | **0.045** | **0.88** |
| ***Chordc1*** | **826** | **537** | **0.65** | **0.0026** | **0.35** |
| ***Timp4*** | **685** | **446** | **0.65** | **0.0032** | **0.38** |
| ***Nrxn2*** | **101** | **66** | **0.65** | **0.012** | **0.59** |
| ***D9Ertd402e*** | **334** | **218** | **0.65** | **0.0045** | **0.40** |
| ***Pdpn*** | **389** | **254** | **0.65** | **0.0031** | **0.38** |
| ***Neat1*** | **7624** | **4977** | **0.65** | **0.0044** | **0.40** |
| ***Uqcc*** | **968** | **632** | **0.65** | **0.047** | **0.89** |
| ***Trappc3*** | **2207** | **1447** | **0.66** | **0.015** | **0.63** |
| ***Ap3m2*** | **184** | **121** | **0.66** | **0.038** | **0.85** |
| ***Dll4*** | **649** | **427** | **0.66** | **0.0058** | **0.45** |
| ***Cd2bp2*** | **350** | **231** | **0.66** | **0.0039** | **0.39** |
| ***Gcat*** | **360** | **238** | **0.66** | **0.0050** | **0.41** |
| ***Cyth3*** | **1164** | **770** | **0.66** | **0.0051** | **0.41** |
| ***Bcl7b*** | **1489** | **986** | **0.66** | **0.016** | **0.64** |
| ***Snrpc*** | **226** | **149** | **0.66** | **0.020** | **0.72** |
| ***A530020G20Rik*** | **470** | **312** | **0.66** | **0.0034** | **0.38** |
| ***Thrsp*** | **10391** | **6889** | **0.66** | **0.012** | **0.58** |
| ***Hspd1*** | **2523** | **1675** | **0.66** | **0.0017** | **0.28** |
| ***Gnb5*** | **242** | **161** | **0.66** | **0.0076** | **0.51** |
| ***Aacs*** | **381** | **253** | **0.67** | **0.025** | **0.77** |
| ***Rfesd*** | **77** | **51** | **0.67** | **0.028** | **0.78** |
| ***1500017E21Rik*** | **159** | **106** | **0.67** | **0.014** | **0.61** |
| ***Slc2a13*** | **209** | **139** | **0.67** | **0.0069** | **0.48** |
| ***Pp2d1*** | **60** | **40** | **0.67** | **0.041** | **0.86** |
| ***Hoxd9*** | **50** | **34** | **0.67** | **0.045** | **0.88** |
| ***Wdr18*** | **171** | **115** | **0.67** | **0.013** | **0.59** |
| ***Bid*** | **690** | **462** | **0.67** | **0.027** | **0.77** |
| ***Bak1*** | **749** | **502** | **0.67** | **0.045** | **0.88** |
| ***Snhg10*** | **128** | **85** | **0.67** | **0.018** | **0.69** |
| ***Anxa9*** | **264** | **177** | **0.67** | **0.0060** | **0.45** |
| ***B3galt2*** | **2965** | **1991** | **0.67** | **0.0031** | **0.38** |
| ***Stk19*** | **424** | **285** | **0.67** | **0.011** | **0.56** |
| ***Josd2*** | **157** | **106** | **0.67** | **0.014** | **0.61** |
| ***Glb1l*** | **191** | **129** | **0.67** | **0.0095** | **0.55** |
| ***Fahd2a*** | **56** | **38** | **0.67** | **0.048** | **0.89** |
| ***Dlst*** | **4560** | **3072** | **0.67** | **0.0034** | **0.38** |
| ***Fmo1*** | **267** | **180** | **0.67** | **0.0095** | **0.55** |
| ***Flcn*** | **246** | **166** | **0.67** | **0.0081** | **0.51** |
| ***Steap2*** | **144** | **97** | **0.68** | **0.010** | **0.56** |
| ***Ttc23*** | **111** | **75** | **0.68** | **0.026** | **0.77** |
| ***Pnpla2*** | **22446** | **15211** | **0.68** | **0.0032** | **0.38** |
| ***2810002N01Rik*** | **1051** | **714** | **0.68** | **0.010** | **0.55** |
| ***Fbln1*** | **370** | **252** | **0.68** | **0.045** | **0.88** |
| ***Tspo*** | **21361** | **14530** | **0.68** | **0.0080** | **0.51** |
| ***Mlh1*** | **813** | **553** | **0.68** | **0.011** | **0.56** |
| ***Zfp68*** | **107** | **73** | **0.68** | **0.025** | **0.76** |
| ***Usp14*** | **508** | **346** | **0.68** | **0.0081** | **0.51** |
| ***Rpgrip1*** | **549** | **374** | **0.68** | **0.010** | **0.55** |
| ***Clcn2*** | **133** | **90** | **0.68** | **0.018** | **0.68** |
| ***Zbtb26*** | **118** | **80** | **0.68** | **0.023** | **0.74** |
| ***Kdm2a*** | **259** | **177** | **0.68** | **0.045** | **0.88** |
| ***Xrcc5*** | **220** | **150** | **0.68** | **0.0097** | **0.55** |
| ***2610020H08Rik*** | **63** | **43** | **0.68** | **0.045** | **0.88** |
| ***Gpm6a*** | **1087** | **743** | **0.68** | **0.0051** | **0.41** |
| ***Bloc1s3*** | **3375** | **2308** | **0.68** | **0.0047** | **0.40** |
| ***Mmachc*** | **213** | **146** | **0.68** | **0.022** | **0.73** |
| ***Pex16*** | **279** | **191** | **0.68** | **0.014** | **0.61** |
| ***Ptpn9*** | **104** | **71** | **0.68** | **0.021** | **0.72** |
| ***Nat8l*** | **131** | **90** | **0.69** | **0.023** | **0.74** |
| ***Hoxa5*** | **171** | **117** | **0.69** | **0.017** | **0.67** |
| ***Aco1*** | **468** | **322** | **0.69** | **0.0070** | **0.49** |
| ***Pard6g*** | **376** | **258** | **0.69** | **0.0083** | **0.52** |
| ***Rab14*** | **4517** | **3107** | **0.69** | **0.0047** | **0.40** |
| ***Fxyd3*** | **67** | **46** | **0.69** | **0.041** | **0.86** |
| ***Timmdc1*** | **878** | **606** | **0.69** | **0.029** | **0.80** |
| ***Larp1*** | **1249** | **862** | **0.69** | **0.035** | **0.84** |
| ***Lat*** | **159** | **110** | **0.69** | **0.015** | **0.63** |
| ***Klf16*** | **180** | **124** | **0.69** | **0.045** | **0.88** |
| ***Fbxo7*** | **112** | **77** | **0.69** | **0.028** | **0.78** |
| ***Cpt2*** | **1714** | **1185** | **0.69** | **0.0077** | **0.51** |
| ***Abcc1*** | **955** | **661** | **0.69** | **0.033** | **0.83** |
| ***Fam192a*** | **730** | **505** | **0.69** | **0.033** | **0.83** |
| ***Gde1*** | **3037** | **2105** | **0.69** | **0.0097** | **0.55** |
| ***Sh3bp4*** | **110** | **77** | **0.69** | **0.034** | **0.84** |
| ***Phyh*** | **3423** | **2377** | **0.69** | **0.0056** | **0.44** |
| ***Ablim3*** | **260** | **180** | **0.69** | **0.012** | **0.59** |
| ***C2*** | **2189** | **1522** | **0.70** | **0.0058** | **0.45** |
| ***1810043G02Rik*** | **108** | **75** | **0.70** | **0.044** | **0.88** |
| ***Bhlhb9*** | **258** | **181** | **0.70** | **0.014** | **0.61** |
| ***Zfp637*** | **295** | **206** | **0.70** | **0.018** | **0.69** |
| ***Ano1*** | **358** | **251** | **0.70** | **0.012** | **0.59** |
| ***Ndufv2*** | **1475** | **1032** | **0.70** | **0.042** | **0.86** |
| ***Ppp1r3c*** | **1341** | **938** | **0.70** | **0.030** | **0.80** |
| ***Gbe1*** | **510** | **357** | **0.70** | **0.016** | **0.65** |
| ***Add1*** | **1145** | **803** | **0.70** | **0.048** | **0.89** |
| ***Crtap*** | **1703** | **1195** | **0.70** | **0.034** | **0.84** |
| ***Fgf10*** | **1721** | **1208** | **0.70** | **0.014** | **0.61** |
| ***Slc2a4*** | **874** | **613** | **0.70** | **0.011** | **0.56** |
| ***F11r*** | **816** | **574** | **0.70** | **0.0082** | **0.51** |
| ***Gipc1*** | **890** | **626** | **0.70** | **0.035** | **0.84** |
| ***Prlr*** | **313** | **220** | **0.70** | **0.015** | **0.62** |
| ***Maml1*** | **390** | **274** | **0.70** | **0.018** | **0.69** |
| ***Dpp4*** | **1194** | **841** | **0.70** | **0.011** | **0.56** |
| ***Slc16a7*** | **689** | **485** | **0.70** | **0.047** | **0.89** |
| ***Usp1*** | **747** | **527** | **0.71** | **0.015** | **0.63** |
| ***2610034B18Rik*** | **284** | **201** | **0.71** | **0.030** | **0.81** |
| ***Stmn2*** | **162** | **114** | **0.71** | **0.025** | **0.76** |
| ***H2-Ke6*** | **350** | **248** | **0.71** | **0.049** | **0.90** |
| ***Mterfd3*** | **165** | **117** | **0.71** | **0.026** | **0.77** |
| ***Mrps15*** | **1718** | **1218** | **0.71** | **0.0093** | **0.55** |
| ***Dtd1*** | **234** | **166** | **0.71** | **0.024** | **0.75** |
| ***Lrig3*** | **116** | **83** | **0.71** | **0.039** | **0.86** |
| ***Lancl2*** | **102** | **73** | **0.71** | **0.044** | **0.88** |
| ***Bphl*** | **2949** | **2098** | **0.71** | **0.017** | **0.67** |
| ***Vezf1*** | **1241** | **883** | **0.71** | **0.012** | **0.59** |
| ***Txlng*** | **375** | **267** | **0.71** | **0.018** | **0.68** |
| ***Tmem29*** | **291** | **207** | **0.71** | **0.017** | **0.66** |
| ***Hspa2*** | **171** | **122** | **0.71** | **0.024** | **0.76** |
| ***Nbl1*** | **80** | **57** | **0.71** | **0.036** | **0.84** |
| ***Mcee*** | **668** | **476** | **0.71** | **0.021** | **0.72** |
| ***Slc25a25*** | **152** | **109** | **0.71** | **0.030** | **0.81** |
| ***2810013P06Rik*** | **117** | **84** | **0.72** | **0.030** | **0.81** |
| ***E4f1*** | **173** | **124** | **0.72** | **0.024** | **0.75** |
| ***Cenpw*** | **352** | **252** | **0.72** | **0.024** | **0.76** |
| ***Tmem147*** | **1081** | **775** | **0.72** | **0.014** | **0.61** |
| ***Cd151*** | **4680** | **3358** | **0.72** | **0.020** | **0.71** |
| ***Ddx54*** | **173** | **124** | **0.72** | **0.034** | **0.84** |
| ***Nrip1*** | **743** | **533** | **0.72** | **0.018** | **0.69** |
| ***Antxr2*** | **864** | **620** | **0.72** | **0.012** | **0.59** |
| ***8430406I07Rik*** | **161** | **116** | **0.72** | **0.042** | **0.86** |
| ***Arl4a*** | **6163** | **4428** | **0.72** | **0.017** | **0.67** |
| ***Cbfa2t3*** | **282** | **203** | **0.72** | **0.029** | **0.79** |
| ***Gpr133*** | **1477** | **1065** | **0.72** | **0.016** | **0.65** |
| ***Atp2b4*** | **2686** | **1938** | **0.72** | **0.012** | **0.59** |
| ***Ring1*** | **404** | **292** | **0.72** | **0.020** | **0.72** |
| ***Thbd*** | **3912** | **2827** | **0.72** | **0.022** | **0.74** |
| ***Ppl*** | **268** | **193** | **0.72** | **0.019** | **0.70** |
| ***Fam175a*** | **158** | **114** | **0.72** | **0.047** | **0.89** |
| ***Ahr*** | **221** | **160** | **0.72** | **0.039** | **0.86** |
| ***Gtl3*** | **470** | **340** | **0.72** | **0.024** | **0.75** |
| ***G0s2*** | **26566** | **19247** | **0.72** | **0.017** | **0.66** |
| ***4930420K17Rik*** | **913** | **662** | **0.72** | **0.033** | **0.83** |
| ***Vkorc1*** | **779** | **565** | **0.73** | **0.022** | **0.73** |
| ***Tbc1d4*** | **357** | **259** | **0.73** | **0.024** | **0.75** |
| ***Oxnad1*** | **531** | **386** | **0.73** | **0.033** | **0.84** |
| ***Zfp35*** | **244** | **177** | **0.73** | **0.032** | **0.83** |
| ***Ppa2*** | **594** | **433** | **0.73** | **0.029** | **0.80** |
| ***Hist2h3c1*** | **394** | **287** | **0.73** | **0.040** | **0.86** |
| ***Vps13c*** | **502** | **366** | **0.73** | **0.028** | **0.78** |
| ***Brd8*** | **321** | **234** | **0.73** | **0.029** | **0.79** |
| ***Frmd6*** | **998** | **730** | **0.73** | **0.039** | **0.86** |
| ***Dguok*** | **715** | **523** | **0.73** | **0.026** | **0.77** |
| ***Ebf1*** | **902** | **660** | **0.73** | **0.026** | **0.77** |
| ***Ccl27a*** | **166** | **122** | **0.73** | **0.050** | **0.90** |
| ***Mccc1*** | **1580** | **1161** | **0.74** | **0.023** | **0.74** |
| ***Gpsm1*** | **225** | **165** | **0.74** | **0.034** | **0.84** |
| ***2210016L21Rik*** | **379** | **279** | **0.74** | **0.041** | **0.86** |
| ***Med1*** | **330** | **243** | **0.74** | **0.041** | **0.86** |
| ***Dpm1*** | **2535** | **1869** | **0.74** | **0.038** | **0.85** |
| ***Heatr5b*** | **713** | **526** | **0.74** | **0.033** | **0.83** |
| ***Scamp3*** | **1821** | **1345** | **0.74** | **0.035** | **0.84** |
| ***Ndufa12*** | **2314** | **1709** | **0.74** | **0.027** | **0.77** |
| ***Klhl2*** | **951** | **703** | **0.74** | **0.027** | **0.77** |
| ***Siva1*** | **909** | **672** | **0.74** | **0.033** | **0.84** |
| ***Pdzd2*** | **538** | **398** | **0.74** | **0.034** | **0.84** |
| ***Bmper*** | **223** | **165** | **0.74** | **0.045** | **0.88** |
| ***Alkbh7*** | **310** | **230** | **0.74** | **0.038** | **0.85** |
| ***A430018G15Rik*** | **473** | **350** | **0.74** | **0.047** | **0.89** |
| ***Kdr*** | **392** | **290** | **0.74** | **0.038** | **0.85** |
| ***Rab38*** | **213** | **158** | **0.74** | **0.049** | **0.90** |
| ***Sccpdh*** | **1016** | **753** | **0.74** | **0.031** | **0.82** |
| ***Pdha1*** | **8835** | **6556** | **0.74** | **0.034** | **0.84** |
| ***Il17re*** | **196** | **145** | **0.74** | **0.041** | **0.86** |
| ***Edn1*** | **423** | **315** | **0.74** | **0.042** | **0.87** |
| ***Sult1a1*** | **8039** | **5988** | **0.74** | **0.032** | **0.83** |
| ***Prkci*** | **633** | **472** | **0.75** | **0.038** | **0.85** |
| ***Kif13a*** | **557** | **416** | **0.75** | **0.037** | **0.85** |
| ***Fbxo33*** | **382** | **285** | **0.75** | **0.050** | **0.90** |
| ***Gadd45gip1*** | **1716** | **1284** | **0.75** | **0.041** | **0.86** |
| ***Acadsb*** | **368** | **275** | **0.75** | **0.046** | **0.89** |
| ***Mcc*** | **268** | **201** | **0.75** | **0.050** | **0.90** |
| ***Fam125b*** | **372** | **279** | **0.75** | **0.043** | **0.88** |
| ***Ppapdc2*** | **759** | **570** | **0.75** | **0.040** | **0.86** |
| ***Ap3d1*** | **2402** | **1806** | **0.75** | **0.033** | **0.83** |
| ***Fth1*** | **32056** | **24132** | **0.75** | **0.023** | **0.74** |
| ***Bckdhb*** | **4294** | **3242** | **0.75** | **0.048** | **0.89** |
| ***Lpar6*** | **590** | **446** | **0.76** | **0.040** | **0.86** |
| ***Phkb*** | **1757** | **1329** | **0.76** | **0.041** | **0.86** |
| ***Efna5*** | **536** | **406** | **0.76** | **0.048** | **0.89** |
| ***1700034H14Rik*** | **384** | **291** | **0.76** | **0.048** | **0.89** |
| ***Hrsp12*** | **1420** | **1076** | **0.76** | **0.042** | **0.86** |
| ***Rtn4rl1*** | **591** | **448** | **0.76** | **0.041** | **0.86** |
| ***Mgst1*** | **412** | **313** | **0.76** | **0.050** | **0.90** |
| ***Camk2d*** | **1644** | **1250** | **0.76** | **0.039** | **0.86** |
| ***Rab3gap1*** | **468** | **357** | **0.76** | **0.046** | **0.89** |
| ***Sptlc1*** | **1045** | **798** | **0.76** | **0.047** | **0.89** |
| ***Fam120c*** | **464** | **354** | **0.76** | **0.049** | **0.90** |
| ***Arid2*** | **827** | **634** | **0.77** | **0.049** | **0.90** |
| ***Agpat6*** | **2245** | **1730** | **0.77** | **0.048** | **0.89** |
| ***Cdkn2b*** | **606** | **783** | **1.29** | **0.050** | **0.90** |
| ***Ubtd1*** | **412** | **535** | **1.30** | **0.044** | **0.88** |
| ***Prelp*** | **5110** | **6675** | **1.31** | **0.037** | **0.85** |
| ***Pmp22*** | **494** | **648** | **1.31** | **0.050** | **0.90** |
| ***Sh3pxd2b*** | **1412** | **1853** | **1.31** | **0.041** | **0.86** |
| ***Lpgat1*** | **7832** | **10297** | **1.31** | **0.034** | **0.84** |
| ***Emb*** | **414** | **545** | **1.32** | **0.045** | **0.88** |
| ***Psmb2*** | **996** | **1311** | **1.32** | **0.036** | **0.84** |
| ***Fam102b*** | **1802** | **2375** | **1.32** | **0.038** | **0.85** |
| ***Mrpl11*** | **657** | **866** | **1.32** | **0.046** | **0.89** |
| ***Unc93b1*** | **940** | **1241** | **1.32** | **0.042** | **0.86** |
| ***Peg10*** | **332** | **439** | **1.32** | **0.050** | **0.90** |
| ***Rhoa*** | **3346** | **4431** | **1.32** | **0.040** | **0.86** |
| ***Rhob*** | **881** | **1167** | **1.32** | **0.037** | **0.85** |
| ***Rassf4*** | **623** | **826** | **1.32** | **0.034** | **0.84** |
| ***Elovl6*** | **317** | **420** | **1.33** | **0.040** | **0.86** |
| ***Lss*** | **482** | **641** | **1.33** | **0.033** | **0.83** |
| ***Rars*** | **976** | **1298** | **1.33** | **0.049** | **0.90** |
| ***Ctsb*** | **23358** | **31086** | **1.33** | **0.025** | **0.77** |
| ***Cybb*** | **477** | **635** | **1.33** | **0.042** | **0.86** |
| ***Rtn2*** | **1508** | **2011** | **1.33** | **0.040** | **0.86** |
| ***Hspb2*** | **369** | **492** | **1.33** | **0.039** | **0.86** |
| ***1810055G02Rik*** | **410** | **547** | **1.33** | **0.041** | **0.86** |
| ***AI662270*** | **336** | **449** | **1.34** | **0.042** | **0.86** |
| ***Dnmt3a*** | **538** | **720** | **1.34** | **0.028** | **0.78** |
| ***Gsto1*** | **1168** | **1564** | **1.34** | **0.035** | **0.84** |
| ***Col5a2*** | **647** | **866** | **1.34** | **0.045** | **0.88** |
| ***Itpripl2*** | **564** | **755** | **1.34** | **0.027** | **0.77** |
| ***Slc45a4*** | **207** | **277** | **1.34** | **0.049** | **0.90** |
| ***N4bp1*** | **207** | **278** | **1.34** | **0.044** | **0.88** |
| ***Actg1*** | **3880** | **5217** | **1.34** | **0.026** | **0.77** |
| ***Sec11c*** | **480** | **646** | **1.35** | **0.035** | **0.84** |
| ***Frmd8*** | **370** | **498** | **1.35** | **0.047** | **0.89** |
| ***P4hb*** | **3169** | **4267** | **1.35** | **0.024** | **0.75** |
| ***Tpcn1*** | **327** | **440** | **1.35** | **0.048** | **0.89** |
| ***Ikbip*** | **269** | **362** | **1.35** | **0.039** | **0.86** |
| ***Cstb*** | **3381** | **4554** | **1.35** | **0.018** | **0.69** |
| ***Gfpt1*** | **208** | **281** | **1.35** | **0.026** | **0.77** |
| ***Tgs1*** | **169** | **229** | **1.35** | **0.050** | **0.90** |
| ***C1qtnf1*** | **422** | **571** | **1.35** | **0.045** | **0.88** |
| ***Nkd1*** | **1915** | **2598** | **1.36** | **0.026** | **0.77** |
| ***H2-Q4*** | **685** | **930** | **1.36** | **0.032** | **0.83** |
| ***Acot13*** | **405** | **551** | **1.36** | **0.030** | **0.81** |
| ***Tbl1xr1*** | **319** | **433** | **1.36** | **0.035** | **0.84** |
| ***Plekhh2*** | **205** | **278** | **1.36** | **0.036** | **0.84** |
| ***Sipa1l1*** | **240** | **327** | **1.36** | **0.029** | **0.80** |
| ***Vhl*** | **152** | **207** | **1.36** | **0.047** | **0.89** |
| ***Itga4*** | **145** | **197** | **1.36** | **0.047** | **0.89** |
| ***Cd84*** | **138** | **188** | **1.36** | **0.046** | **0.89** |
| ***Txlna*** | **163** | **223** | **1.37** | **0.037** | **0.85** |
| ***Hspg2*** | **1122** | **1533** | **1.37** | **0.020** | **0.71** |
| ***Soat1*** | **198** | **270** | **1.37** | **0.038** | **0.85** |
| ***Sla*** | **171** | **234** | **1.37** | **0.043** | **0.88** |
| ***Coro1b*** | **431** | **589** | **1.37** | **0.027** | **0.77** |
| ***Tspan18*** | **170** | **233** | **1.37** | **0.027** | **0.77** |
| ***Atp8b1*** | **278** | **381** | **1.37** | **0.036** | **0.84** |
| ***Dynlrb1*** | **4885** | **6698** | **1.37** | **0.033** | **0.83** |
| ***Snrpd3*** | **1904** | **2611** | **1.37** | **0.014** | **0.61** |
| ***Heph*** | **214** | **294** | **1.37** | **0.031** | **0.82** |
| ***Pycard*** | **224** | **308** | **1.37** | **0.023** | **0.74** |
| ***Clec4a2*** | **358** | **492** | **1.37** | **0.019** | **0.70** |
| ***Ppp1cc*** | **237** | **326** | **1.38** | **0.027** | **0.77** |
| ***Ift20*** | **122** | **168** | **1.38** | **0.047** | **0.89** |
| ***Inf2*** | **210** | **289** | **1.38** | **0.033** | **0.83** |
| ***C1qb*** | **3398** | **4688** | **1.38** | **0.019** | **0.70** |
| ***Mfge8*** | **13118** | **18108** | **1.38** | **0.017** | **0.66** |
| ***Dhx8*** | **130** | **180** | **1.38** | **0.039** | **0.86** |
| ***Pde7a*** | **490** | **677** | **1.38** | **0.024** | **0.76** |
| ***Xpot*** | **510** | **705** | **1.38** | **0.018** | **0.68** |
| ***Nek9*** | **191** | **264** | **1.38** | **0.033** | **0.84** |
| ***Sgta*** | **878** | **1215** | **1.38** | **0.028** | **0.78** |
| ***2700029M09Rik*** | **111** | **153** | **1.39** | **0.042** | **0.86** |
| ***Emc7*** | **333** | **463** | **1.39** | **0.028** | **0.78** |
| ***Gltp*** | **432** | **599** | **1.39** | **0.019** | **0.70** |
| ***Tnip2*** | **96** | **133** | **1.39** | **0.043** | **0.88** |
| ***Kcnab2*** | **163** | **226** | **1.39** | **0.021** | **0.72** |
| ***Rgs2*** | **281** | **391** | **1.39** | **0.044** | **0.88** |
| ***Aldh18a1*** | **84** | **117** | **1.39** | **0.049** | **0.90** |
| ***Gm14005*** | **2080** | **2892** | **1.39** | **0.015** | **0.62** |
| ***Svil*** | **331** | **461** | **1.39** | **0.043** | **0.87** |
| ***Reps2*** | **122** | **169** | **1.39** | **0.039** | **0.86** |
| ***Arfgap3*** | **84** | **117** | **1.39** | **0.047** | **0.89** |
| ***Ctsc*** | **654** | **911** | **1.39** | **0.026** | **0.77** |
| ***H2-K2*** | **159** | **222** | **1.39** | **0.031** | **0.82** |
| ***Lyz1*** | **16116** | **22541** | **1.40** | **0.011** | **0.56** |
| ***Man1c1*** | **272** | **382** | **1.40** | **0.015** | **0.63** |
| ***Dbndd2*** | **134** | **188** | **1.40** | **0.049** | **0.90** |
| ***Dgkq*** | **85** | **119** | **1.40** | **0.042** | **0.86** |
| ***C3ar1*** | **964** | **1354** | **1.40** | **0.014** | **0.61** |
| ***H2-K1*** | **3231** | **4539** | **1.40** | **0.020** | **0.72** |
| ***Mfn1*** | **74** | **104** | **1.41** | **0.036** | **0.84** |
| ***Nudt19*** | **843** | **1185** | **1.41** | **0.019** | **0.70** |
| ***Tlr7*** | **80** | **113** | **1.41** | **0.033** | **0.83** |
| ***Gfer*** | **295** | **415** | **1.41** | **0.025** | **0.76** |
| ***Rnf115*** | **327** | **461** | **1.41** | **0.029** | **0.80** |
| ***Uvrag*** | **528** | **743** | **1.41** | **0.015** | **0.63** |
| ***Gnpat*** | **966** | **1362** | **1.41** | **0.019** | **0.70** |
| ***Slc19a1*** | **183** | **258** | **1.41** | **0.029** | **0.80** |
| ***Bank1*** | **98** | **138** | **1.41** | **0.045** | **0.88** |
| ***Nfkb2*** | **77** | **109** | **1.41** | **0.041** | **0.86** |
| ***Ywhaq*** | **355** | **501** | **1.41** | **0.019** | **0.70** |
| ***Lpxn*** | **483** | **683** | **1.41** | **0.012** | **0.59** |
| ***Cytip*** | **150** | **213** | **1.41** | **0.020** | **0.72** |
| ***Itgb2*** | **314** | **445** | **1.42** | **0.011** | **0.56** |
| ***Ahsa2*** | **579** | **820** | **1.42** | **0.014** | **0.61** |
| ***Nceh1*** | **159** | **225** | **1.42** | **0.046** | **0.89** |
| ***Fam198b*** | **803** | **1137** | **1.42** | **0.011** | **0.56** |
| ***Chfr*** | **114** | **161** | **1.42** | **0.025** | **0.76** |
| ***Psmb10*** | **1237** | **1755** | **1.42** | **0.0099** | **0.55** |
| ***Mob3a*** | **123** | **174** | **1.42** | **0.017** | **0.67** |
| ***Eif4a3*** | **994** | **1413** | **1.42** | **0.027** | **0.77** |
| ***Arhgef2*** | **268** | **380** | **1.42** | **0.011** | **0.56** |
| ***Maff*** | **206** | **293** | **1.42** | **0.013** | **0.59** |
| ***Tmed9*** | **273** | **388** | **1.42** | **0.042** | **0.86** |
| ***Nedd8*** | **6656** | **9472** | **1.42** | **0.0080** | **0.51** |
| ***Cnpy4*** | **180** | **257** | **1.42** | **0.024** | **0.75** |
| ***Higd2a*** | **2693** | **3834** | **1.42** | **0.0064** | **0.45** |
| ***Tmem181c-ps*** | **99** | **142** | **1.42** | **0.044** | **0.88** |
| ***Serpina3c*** | **1124** | **1601** | **1.42** | **0.036** | **0.84** |
| ***Gmppb*** | **213** | **304** | **1.43** | **0.014** | **0.61** |
| ***Gtf3a*** | **144** | **205** | **1.43** | **0.047** | **0.89** |
| ***Minpp1*** | **734** | **1047** | **1.43** | **0.049** | **0.90** |
| ***Sdc1*** | **106** | **151** | **1.43** | **0.022** | **0.74** |
| ***Arhgap39*** | **65** | **93** | **1.43** | **0.050** | **0.90** |
| ***Gpr56*** | **277** | **395** | **1.43** | **0.017** | **0.66** |
| ***Tm4sf5*** | **81** | **116** | **1.43** | **0.032** | **0.83** |
| ***Brms1*** | **99** | **142** | **1.43** | **0.027** | **0.77** |
| ***0610039K10Rik*** | **123** | **177** | **1.44** | **0.023** | **0.74** |
| ***Traf2*** | **68** | **98** | **1.44** | **0.035** | **0.84** |
| ***0610007N19Rik*** | **249** | **358** | **1.44** | **0.039** | **0.86** |
| ***Hist1h2ao*** | **87** | **125** | **1.44** | **0.035** | **0.84** |
| ***Ece2*** | **54** | **77** | **1.44** | **0.039** | **0.86** |
| ***Uso1*** | **440** | **633** | **1.44** | **0.014** | **0.61** |
| ***Stk24*** | **115** | **166** | **1.44** | **0.047** | **0.89** |
| ***Fam26f*** | **41** | **58** | **1.44** | **0.048** | **0.89** |
| ***Elovl1*** | **141** | **203** | **1.44** | **0.029** | **0.79** |
| ***Athl1*** | **118** | **171** | **1.44** | **0.021** | **0.72** |
| ***Hsd17b4*** | **828** | **1195** | **1.44** | **0.0097** | **0.55** |
| ***Lonrf3*** | **57** | **82** | **1.45** | **0.037** | **0.85** |
| ***Ddx46*** | **262** | **379** | **1.45** | **0.013** | **0.59** |
| ***AB124611*** | **79** | **115** | **1.45** | **0.023** | **0.75** |
| ***Zfp280b*** | **119** | **172** | **1.45** | **0.013** | **0.59** |
| ***Znrf2*** | **165** | **238** | **1.45** | **0.027** | **0.77** |
| ***Prkch*** | **102** | **148** | **1.45** | **0.026** | **0.77** |
| ***Csf2rb2*** | **523** | **760** | **1.45** | **0.0083** | **0.51** |
| ***Adcy7*** | **249** | **362** | **1.45** | **0.0087** | **0.53** |
| ***Acox3*** | **192** | **279** | **1.45** | **0.016** | **0.65** |
| ***H1fx*** | **63** | **91** | **1.45** | **0.034** | **0.84** |
| ***Nup210*** | **56** | **81** | **1.46** | **0.038** | **0.85** |
| ***Lrp12*** | **112** | **162** | **1.46** | **0.031** | **0.82** |
| ***Cwf19l1*** | **112** | **163** | **1.46** | **0.039** | **0.86** |
| ***Uimc1*** | **68** | **99** | **1.46** | **0.043** | **0.87** |
| ***Pvrl3*** | **50** | **73** | **1.46** | **0.040** | **0.86** |
| ***Sec61b*** | **990** | **1444** | **1.46** | **0.050** | **0.90** |
| ***Fdx1*** | **287** | **418** | **1.46** | **0.018** | **0.68** |
| ***Slc48a1*** | **1794** | **2619** | **1.46** | **0.012** | **0.59** |
| ***Hrasls*** | **59** | **86** | **1.46** | **0.044** | **0.88** |
| ***Sycp3*** | **336** | **491** | **1.46** | **0.025** | **0.77** |
| ***Tubgcp3*** | **85** | **125** | **1.46** | **0.044** | **0.88** |
| ***Fgf1*** | **642** | **939** | **1.46** | **0.0090** | **0.54** |
| ***Cln6*** | **163** | **239** | **1.46** | **0.015** | **0.63** |
| ***Ankmy2*** | **66** | **97** | **1.46** | **0.028** | **0.78** |
| ***Asah1*** | **710** | **1040** | **1.46** | **0.0040** | **0.39** |
| ***Agpat4*** | **99** | **146** | **1.47** | **0.013** | **0.60** |
| ***Lif*** | **45** | **66** | **1.47** | **0.050** | **0.90** |
| ***Apobr*** | **257** | **377** | **1.47** | **0.0077** | **0.51** |
| ***Bcl3*** | **127** | **186** | **1.47** | **0.012** | **0.59** |
| ***Fkbp2*** | **156** | **229** | **1.47** | **0.011** | **0.56** |
| ***Irf5*** | **50** | **74** | **1.47** | **0.036** | **0.84** |
| ***Phf15*** | **89** | **131** | **1.47** | **0.032** | **0.83** |
| ***Il1r1*** | **88** | **130** | **1.47** | **0.017** | **0.66** |
| ***Fam122b*** | **92** | **136** | **1.47** | **0.014** | **0.61** |
| ***Cdk20*** | **81** | **120** | **1.47** | **0.026** | **0.77** |
| ***Ap3m1*** | **211** | **311** | **1.47** | **0.0091** | **0.54** |
| ***Serping1*** | **6242** | **9204** | **1.47** | **0.0060** | **0.45** |
| ***Tpcn2*** | **339** | **501** | **1.48** | **0.0062** | **0.45** |
| ***Rps6ka4*** | **261** | **385** | **1.48** | **0.0061** | **0.45** |
| ***Tmem86a*** | **172** | **254** | **1.48** | **0.013** | **0.59** |
| ***Ikbke*** | **215** | **318** | **1.48** | **0.011** | **0.57** |
| ***Pfn1*** | **3125** | **4620** | **1.48** | **0.0044** | **0.40** |
| ***Cd209b*** | **83** | **123** | **1.48** | **0.022** | **0.73** |
| ***Scyl1*** | **467** | **692** | **1.48** | **0.022** | **0.73** |
| ***Zbtb38*** | **41** | **61** | **1.48** | **0.044** | **0.88** |
| ***Cd300a*** | **120** | **178** | **1.48** | **0.048** | **0.89** |
| ***Auh*** | **417** | **619** | **1.49** | **0.050** | **0.90** |
| ***Ryr1*** | **45** | **66** | **1.49** | **0.048** | **0.89** |
| ***Man1a*** | **173** | **257** | **1.49** | **0.020** | **0.72** |
| ***Prrc2b*** | **308** | **459** | **1.49** | **0.014** | **0.61** |
| ***Plod2*** | **54** | **81** | **1.49** | **0.034** | **0.84** |
| ***Lipa*** | **1272** | **1904** | **1.50** | **0.0048** | **0.40** |
| ***Sh2d1b1*** | **43** | **64** | **1.50** | **0.050** | **0.90** |
| ***Cd300lb*** | **219** | **328** | **1.50** | **0.028** | **0.78** |
| ***Lcp1*** | **793** | **1189** | **1.50** | **0.0034** | **0.38** |
| ***Tnip3*** | **110** | **165** | **1.50** | **0.0071** | **0.49** |
| ***Lilrb4*** | **299** | **449** | **1.50** | **0.0048** | **0.40** |
| ***Il3ra*** | **33** | **50** | **1.50** | **0.042** | **0.86** |
| ***Endou*** | **92** | **138** | **1.50** | **0.020** | **0.71** |
| ***Cotl1*** | **558** | **838** | **1.50** | **0.0048** | **0.40** |
| ***Serpina3m*** | **118** | **178** | **1.50** | **0.010** | **0.56** |
| ***Abhd12*** | **103** | **155** | **1.51** | **0.011** | **0.56** |
| ***Slc40a1*** | **159** | **240** | **1.51** | **0.010** | **0.56** |
| ***Adora1*** | **581** | **875** | **1.51** | **0.013** | **0.61** |
| ***Ddah1*** | **108** | **163** | **1.51** | **0.011** | **0.56** |
| ***Slc38a10*** | **820** | **1236** | **1.51** | **0.0044** | **0.40** |
| ***Axl*** | **519** | **783** | **1.51** | **0.0035** | **0.38** |
| ***Scube1*** | **258** | **390** | **1.51** | **0.0050** | **0.41** |
| ***Cog3*** | **52** | **78** | **1.51** | **0.048** | **0.89** |
| ***Erlin1*** | **107** | **161** | **1.51** | **0.050** | **0.90** |
| ***Cacna1d*** | **41** | **62** | **1.51** | **0.027** | **0.77** |
| ***1700047G03Rik*** | **2216** | **3352** | **1.51** | **0.0044** | **0.40** |
| ***Tnip1*** | **345** | **521** | **1.51** | **0.0032** | **0.38** |
| ***Cd200r1*** | **43** | **65** | **1.51** | **0.023** | **0.75** |
| ***A930005H10Rik*** | **123** | **187** | **1.51** | **0.038** | **0.85** |
| ***Ddr1*** | **422** | **640** | **1.51** | **0.0093** | **0.55** |
| ***P2rx7*** | **90** | **137** | **1.52** | **0.0079** | **0.51** |
| ***Ctss*** | **2857** | **4334** | **1.52** | **0.0090** | **0.54** |
| ***Cd300ld*** | **110** | **167** | **1.52** | **0.0073** | **0.50** |
| ***Mtap7*** | **76** | **115** | **1.52** | **0.021** | **0.72** |
| ***Slc6a6*** | **386** | **587** | **1.52** | **0.0032** | **0.38** |
| ***Cdk11b*** | **124** | **190** | **1.53** | **0.0097** | **0.55** |
| ***Dap*** | **880** | **1343** | **1.53** | **0.0037** | **0.38** |
| ***Syk*** | **261** | **399** | **1.53** | **0.0046** | **0.40** |
| ***Lctl*** | **1700** | **2597** | **1.53** | **0.012** | **0.59** |
| ***Cct5*** | **553** | **845** | **1.53** | **0.037** | **0.85** |
| ***C230081A13Rik*** | **357** | **546** | **1.53** | **0.0080** | **0.51** |
| ***Runx2*** | **74** | **113** | **1.53** | **0.015** | **0.62** |
| ***Gm7694*** | **92** | **141** | **1.53** | **0.024** | **0.76** |
| ***Apobec1*** | **67** | **102** | **1.53** | **0.017** | **0.67** |
| ***Park7*** | **554** | **848** | **1.53** | **0.0017** | **0.28** |
| ***Slc36a1*** | **367** | **563** | **1.53** | **0.0025** | **0.34** |
| ***Fam96a*** | **749** | **1150** | **1.53** | **0.0042** | **0.40** |
| ***Ccl6*** | **623** | **957** | **1.54** | **0.016** | **0.64** |
| ***Cd200r4*** | **63** | **97** | **1.54** | **0.0099** | **0.55** |
| ***Sult4a1*** | **50** | **76** | **1.54** | **0.040** | **0.86** |
| ***Arl11*** | **66** | **101** | **1.54** | **0.012** | **0.59** |
| ***Npl*** | **56** | **87** | **1.55** | **0.014** | **0.61** |
| ***Sema7a*** | **47** | **72** | **1.55** | **0.032** | **0.83** |
| ***Lat2*** | **94** | **145** | **1.55** | **0.016** | **0.65** |
| ***Fam189a2*** | **97** | **150** | **1.55** | **0.010** | **0.56** |
| ***Mycbp2*** | **3542** | **5491** | **1.55** | **0.0039** | **0.39** |
| ***Alkbh3*** | **632** | **980** | **1.55** | **0.0095** | **0.55** |
| ***Slamf9*** | **158** | **246** | **1.55** | **0.0049** | **0.40** |
| ***Mbtps2*** | **41** | **64** | **1.56** | **0.019** | **0.70** |
| ***Arhgap25*** | **228** | **356** | **1.56** | **0.0034** | **0.38** |
| ***Cpxm1*** | **297** | **465** | **1.56** | **0.015** | **0.63** |
| ***Ctdp1*** | **34** | **53** | **1.56** | **0.036** | **0.84** |
| ***Hpgds*** | **133** | **208** | **1.57** | **0.0038** | **0.38** |
| ***Hk3*** | **61** | **95** | **1.57** | **0.014** | **0.61** |
| ***Tmem120a*** | **50** | **79** | **1.57** | **0.019** | **0.70** |
| ***Degs2*** | **35** | **55** | **1.57** | **0.044** | **0.88** |
| ***Appl2*** | **102** | **160** | **1.57** | **0.0044** | **0.40** |
| ***Ogfr*** | **38** | **59** | **1.57** | **0.037** | **0.85** |
| ***Lamb3*** | **50** | **78** | **1.58** | **0.013** | **0.59** |
| ***Rps3*** | **3294** | **5189** | **1.58** | **0.0067** | **0.47** |
| ***Nhp2*** | **91** | **144** | **1.58** | **0.0060** | **0.45** |
| ***Tmem179*** | **32** | **51** | **1.58** | **0.025** | **0.76** |
| ***Egr2*** | **43** | **68** | **1.58** | **0.019** | **0.70** |
| ***Galnt6*** | **38** | **60** | **1.59** | **0.027** | **0.77** |
| ***Tmem176b*** | **1360** | **2158** | **1.59** | **0.0073** | **0.50** |
| ***Ccl7*** | **576** | **915** | **1.59** | **0.013** | **0.59** |
| ***Zbp1*** | **64** | **101** | **1.59** | **0.0077** | **0.51** |
| ***Il1rn*** | **136** | **216** | **1.59** | **0.0016** | **0.27** |
| ***2810474O19Rik*** | **85** | **136** | **1.60** | **0.0088** | **0.53** |
| ***Ext2*** | **260** | **414** | **1.60** | **0.00083** | **0.18** |
| ***Rnaseh2a*** | **42** | **67** | **1.60** | **0.021** | **0.72** |
| ***Itgam*** | **211** | **338** | **1.60** | **0.0019** | **0.29** |
| ***Orc5*** | **221** | **355** | **1.60** | **0.0017** | **0.27** |
| ***Ptpn18*** | **61** | **97** | **1.60** | **0.0062** | **0.45** |
| ***Npc2*** | **5475** | **8785** | **1.60** | **0.00028** | **0.11** |
| ***Rpl21*** | **37** | **60** | **1.61** | **0.045** | **0.88** |
| ***Snhg5*** | **100** | **161** | **1.61** | **0.038** | **0.85** |
| ***Slc23a2*** | **247** | **397** | **1.61** | **0.00053** | **0.14** |
| ***Gm6211*** | **166** | **268** | **1.61** | **0.0073** | **0.50** |
| ***P2ry14*** | **84** | **136** | **1.61** | **0.0036** | **0.38** |
| ***Tmem165*** | **589** | **951** | **1.62** | **0.035** | **0.84** |
| ***E130310I04Rik*** | **42** | **68** | **1.62** | **0.0096** | **0.55** |
| ***Bcat1*** | **73** | **118** | **1.62** | **0.0049** | **0.40** |
| ***Ncan*** | **283** | **461** | **1.63** | **0.00052** | **0.14** |
| ***Oxtr*** | **1500** | **2460** | **1.64** | **0.00030** | **0.11** |
| ***Ly9*** | **70** | **115** | **1.64** | **0.0034** | **0.38** |
| ***Tfpi2*** | **261** | **428** | **1.64** | **0.00071** | **0.16** |
| ***Myom3*** | **73** | **120** | **1.65** | **0.0032** | **0.38** |
| ***Hmgb2*** | **197** | **325** | **1.65** | **0.0087** | **0.53** |
| ***Snx18*** | **71** | **116** | **1.65** | **0.010** | **0.56** |
| ***Armcx3*** | **53** | **88** | **1.65** | **0.0076** | **0.51** |
| ***Tssc4*** | **102** | **168** | **1.65** | **0.0015** | **0.26** |
| ***Col6a5*** | **251** | **415** | **1.66** | **0.029** | **0.80** |
| ***Calr*** | **601** | **998** | **1.66** | **0.00025** | **0.10** |
| ***Tmem176a*** | **86** | **142** | **1.66** | **0.0024** | **0.34** |
| ***Tcn2*** | **462** | **766** | **1.66** | **0.041** | **0.86** |
| ***Vasp*** | **136** | **227** | **1.66** | **0.023** | **0.75** |
| ***Tmem88*** | **87** | **145** | **1.67** | **0.040** | **0.86** |
| ***Slamf7*** | **58** | **97** | **1.67** | **0.0035** | **0.38** |
| ***Dnase2a*** | **63** | **105** | **1.67** | **0.0035** | **0.38** |
| ***Btd*** | **32** | **53** | **1.67** | **0.014** | **0.62** |
| ***Ucp2*** | **1167** | **1953** | **1.67** | **8.9E-05** | **0.053** |
| ***Socs3*** | **73** | **123** | **1.68** | **0.0019** | **0.29** |
| ***Atp6v0a1*** | **657** | **1101** | **1.68** | **0.00013** | **0.066** |
| ***Eif3m*** | **360** | **605** | **1.68** | **0.00032** | **0.11** |
| ***Zfand2a*** | **355** | **597** | **1.68** | **0.00045** | **0.13** |
| ***Rcl1*** | **110** | **185** | **1.68** | **0.0028** | **0.37** |
| ***Slc37a2*** | **129** | **217** | **1.68** | **0.00089** | **0.19** |
| ***Atp1a3*** | **43** | **72** | **1.69** | **0.0061** | **0.45** |
| ***Tlr13*** | **50** | **84** | **1.69** | **0.0045** | **0.40** |
| ***Fes*** | **99** | **169** | **1.70** | **0.0012** | **0.24** |
| ***Slamf8*** | **70** | **120** | **1.71** | **0.0021** | **0.30** |
| ***Mrap*** | **716** | **1225** | **1.71** | **0.00011** | **0.062** |
| ***Skp2*** | **59** | **102** | **1.71** | **0.033** | **0.83** |
| ***Ryr2*** | **34** | **58** | **1.71** | **0.024** | **0.75** |
| ***Gpr132*** | **65** | **111** | **1.72** | **0.027** | **0.77** |
| ***Shroom2*** | **53** | **90** | **1.72** | **0.0024** | **0.34** |
| ***Gpr146*** | **408** | **704** | **1.72** | **0.020** | **0.71** |
| ***Banf1*** | **56** | **97** | **1.72** | **0.0059** | **0.45** |
| ***Trem2*** | **349** | **602** | **1.73** | **0.0013** | **0.24** |
| ***Pdgfa*** | **29** | **50** | **1.73** | **0.0099** | **0.55** |
| ***Sep9*** | **189** | **331** | **1.75** | **0.0036** | **0.38** |
| ***Vegfb*** | **3778** | **6611** | **1.75** | **0.025** | **0.77** |
| ***Akt1*** | **1068** | **1874** | **1.75** | **0.0079** | **0.51** |
| ***Sema4d*** | **111** | **196** | **1.77** | **0.0011** | **0.22** |
| ***Evc*** | **37** | **65** | **1.78** | **0.0047** | **0.40** |
| ***Gpnmb*** | **2060** | **3674** | **1.78** | **0.00087** | **0.19** |
| ***1100001G20Rik*** | **1811** | **3242** | **1.79** | **0.00069** | **0.16** |
| ***Gm5617*** | **59** | **106** | **1.79** | **0.031** | **0.82** |
| ***C5ar1*** | **227** | **408** | **1.80** | **0.00061** | **0.15** |
| ***Ncf4*** | **59** | **106** | **1.80** | **0.034** | **0.84** |
| ***Shb*** | **30** | **53** | **1.80** | **0.019** | **0.70** |
| ***Syngr1*** | **114** | **205** | **1.80** | **0.00054** | **0.14** |
| ***Cd99*** | **1161** | **2088** | **1.80** | **0.00092** | **0.19** |
| ***Ptpn7*** | **93** | **166** | **1.80** | **0.00041** | **0.13** |
| ***Dpep2*** | **135** | **243** | **1.80** | **7.1E-05** | **0.050** |
| ***Igsf21*** | **141** | **253** | **1.80** | **0.010** | **0.56** |
| ***Fmn1*** | **32** | **58** | **1.80** | **0.0033** | **0.38** |
| ***Fyb*** | **126** | **227** | **1.80** | **0.0052** | **0.42** |
| ***Cyp2c44*** | **38** | **70** | **1.81** | **0.041** | **0.86** |
| ***Nf1*** | **141** | **254** | **1.81** | **0.016** | **0.65** |
| ***D3Bwg0562e*** | **65** | **118** | **1.81** | **0.0069** | **0.48** |
| ***Mrpl22*** | **82** | **149** | **1.81** | **0.00039** | **0.12** |
| ***Cyp27a1*** | **125** | **227** | **1.82** | **0.00031** | **0.11** |
| ***Rnf128*** | **77** | **140** | **1.82** | **0.00070** | **0.16** |
| ***Atp8b4*** | **96** | **176** | **1.82** | **0.0017** | **0.27** |
| ***Il20rb*** | **34** | **62** | **1.82** | **0.0018** | **0.28** |
| ***H2-Q5*** | **29** | **52** | **1.83** | **0.0076** | **0.51** |
| ***Prrx1*** | **346** | **635** | **1.83** | **0.00073** | **0.17** |
| ***Def8*** | **211** | **388** | **1.84** | **0.00088** | **0.19** |
| ***Acbd4*** | **1441** | **2657** | **1.84** | **0.044** | **0.88** |
| ***Spp1*** | **65** | **119** | **1.84** | **0.00031** | **0.11** |
| ***Polr3d*** | **174** | **323** | **1.85** | **0.0057** | **0.45** |
| ***A630072M18Rik*** | **54** | **102** | **1.87** | **0.00045** | **0.13** |
| ***Xrn2*** | **331** | **618** | **1.87** | **0.021** | **0.72** |
| ***Lbp*** | **6624** | **12404** | **1.87** | **7.2E-06** | **0.0076** |
| ***Itgax*** | **340** | **644** | **1.89** | **0.017** | **0.67** |
| ***Olfml3*** | **44** | **84** | **1.90** | **0.00061** | **0.15** |
| ***St14*** | **30** | **57** | **1.90** | **0.0015** | **0.26** |
| ***Dock2*** | **216** | **411** | **1.90** | **0.0035** | **0.38** |
| ***Stk11*** | **183** | **349** | **1.91** | **0.033** | **0.83** |
| ***Adssl1*** | **325** | **622** | **1.91** | **0.00024** | **0.10** |
| ***A830007P12Rik*** | **41** | **79** | **1.92** | **0.023** | **0.74** |
| ***Pdzk1ip1*** | **57** | **109** | **1.92** | **0.014** | **0.61** |
| ***Lrmp*** | **27** | **52** | **1.93** | **0.0014** | **0.25** |
| ***Calm2*** | **6593** | **12738** | **1.93** | **0.0011** | **0.22** |
| ***Mrc2*** | **169** | **326** | **1.93** | **1.2E-05** | **0.011** |
| ***Mapkapk2*** | **985** | **1906** | **1.94** | **0.026** | **0.77** |
| ***Mcm7*** | **83** | **161** | **1.94** | **0.0040** | **0.39** |
| ***Palld*** | **47** | **91** | **1.96** | **0.0085** | **0.53** |
| ***Lilrb3*** | **69** | **135** | **1.96** | **8.9E-05** | **0.053** |
| ***Mrpl55*** | **54** | **108** | **1.99** | **0.0029** | **0.37** |
| ***Irf8*** | **131** | **262** | **2.00** | **1.2E-05** | **0.011** |
| ***Pcsk4*** | **54** | **109** | **2.02** | **0.041** | **0.86** |
| ***9030619P08Rik*** | **128** | **261** | **2.03** | **0.00022** | **0.10** |
| ***Gla*** | **94** | **191** | **2.04** | **8.6E-06** | **0.0083** |
| ***Trappc6a*** | **95** | **195** | **2.06** | **6.5E-06** | **0.0076** |
| ***Wisp2*** | **439** | **923** | **2.10** | **0.00071** | **0.16** |
| ***Otop1*** | **60** | **127** | **2.11** | **6.1E-05** | **0.045** |
| ***Itgad*** | **37** | **79** | **2.11** | **0.036** | **0.84** |
| ***Rnf185*** | **86** | **184** | **2.13** | **0.012** | **0.58** |
| ***Csprs*** | **38** | **81** | **2.13** | **0.00048** | **0.14** |
| ***Lipf*** | **100** | **216** | **2.16** | **7.3E-06** | **0.0076** |
| ***Hp*** | **47204** | **102374** | **2.17** | **2.9E-08** | **6.5E-05** |
| ***Atad3a*** | **86** | **187** | **2.18** | **0.0064** | **0.45** |
| ***Slc5a7*** | **852** | **1885** | **2.21** | **1.4E-08** | **4.4E-05** |
| ***AF251705*** | **107** | **240** | **2.25** | **0.00021** | **0.094** |
| ***Lrg1*** | **4048** | **9143** | **2.26** | **9.6E-09** | **4.4E-05** |
| ***Darc*** | **107** | **244** | **2.28** | **0.0010** | **0.21** |
| ***Ccdc109b*** | **122** | **286** | **2.34** | **3.0E-08** | **6.5E-05** |
| ***Adprm*** | **24** | **56** | **2.39** | **0.0028** | **0.37** |
| ***Dock8*** | **241** | **584** | **2.43** | **0.032** | **0.83** |
| ***Pop4*** | **53** | **131** | **2.47** | **0.00019** | **0.091** |
| ***Ubd*** | **1174** | **2914** | **2.48** | **8.7E-05** | **0.053** |
| ***Atp6v0d2*** | **108** | **290** | **2.69** | **1.2E-08** | **4.4E-05** |
| ***Cdkl4*** | **52** | **141** | **2.70** | **0.00033** | **0.11** |
| ***Tfr2*** | **50** | **135** | **2.71** | **1.2E-08** | **4.4E-05** |
| ***Saa3*** | **47** | **144** | **3.03** | **0.0082** | **0.51** |
| ***Trdn*** | **30** | **111** | **3.65** | **3.4E-08** | **6.5E-05** |
| ***Tph2*** | **120** | **540** | **4.52** | **0.00021** | **0.094** |
| ***Mest*** | **1663** | **25107** | **15.09** | **1.6E-65** | **2.5E-61** |

eWAT, epididymal white adipose tissue; WT, wildtype mice; pKO, *Mest***^pKO^** mice; FC, fold change.

^a^p-values were calculated using SAM as described in Materials and Methods.

^b^p-values were adjusted for false discovery rate using the Benjamini-Hochberg method.
