# Supplementary material for: Diet-induced adipose tissue expansion is mitigated in mice with a targeted inactivation of mesoderm specific transcript (Mest)
Source: PLoS One. 2017 Jun 22;12(6):e0179879. doi: 10.1371/journal.pone.0179879 (PMC5481029; doi:10.1371/journal.pone.0179879)
Supplement: S3 Table — (DOCX) [file pone.0179879.s004.docx]

**S3 Table: Murine Cohorts and Study Design**

| **Cohort** | **Diet and Age (wks)** | **Genotypes (n)** | **Figures and Tables** |
| --- | --- | --- | --- |
| **Global *Mest* KO** | **(A) W-8; (C) 8-16** | **WT (n=17); pKO (n=10)** | **Fig 2, 5, 6, 7, 8; Table 1, S1, S2** |
| ***Adipoq*-cre *Mest* KO** | **(B) W-8; (C) 8-24** | **WT (n=9); WT-cre (n=5); pFL (n=11); ApKO (n=17)** | **Fig 3** |
| ***Fabp4*-cre *Mest* KO** | **(A) W-8; (C) 8-24** | **WT (n=8); WT-cre (n=11); pFL (n=6); FpKO (n=12)** | **S1 Fig** |
| **GTT and ITT Study** | **(B) W-8; (C) 8-16** | **WT (n=8); pKO (n=7)** | **Fig 4 (A-C)** |
| **Indirect Calorimetry** | **(B) W-8; (C) 8-16** | **WT (n=8); pKO (n=8)** | **Fig 4 (D-J)** |

**W= weaning; (A)=Picolab Rodent Diet 20 (LAB Diet; 13 kcal% fat); (B)=Teklad Global 18% Protein Rodent Diet (Harlan; 18 kcal% fat); (C)=HFD with sucrose; Surwit Diet (D12331, Research Dietsl 58 kcal% fat; 23.26 kJ/g).**
