# Supplementary material for: Diet-induced adipose tissue expansion is mitigated in mice with a targeted inactivation of mesoderm specific transcript (Mest)
Source: PLoS One. 2017 Jun 22;12(6):e0179879. doi: 10.1371/journal.pone.0179879 (PMC5481029; doi:10.1371/journal.pone.0179879)
Supplement: S4 Table — (DOCX) [file pone.0179879.s005.docx]

| **S4 Table. Primer and Probe Sets: qRT-PCR.** | | |  |
| --- | --- | --- | --- |
| **Gene Target** | **Forward Primer (5’-3’)** | **Reverse Primer (5’-3’)** | **TaqMan Probe (5’-3’)** |
| ***Agpat2*** | **CGCGGCCTCGATCGT** | **AAAGCGAAGGCCATACACGTA** | **CGCACCGTGGATAACATGAGCATCAT** |
| ***Atp6v0d2*** | **GCAGACGTTATGTGTCCCATTC** | **TCTCCCTGTCTTCTTTGCTTAGTTC** | **CCGACAGACGCGCTTTAATCATCACTC** |
| ***Bmp3*** | **CAGTTCCCCATGCCAAAGT** | **TTTCCGGCACACAGCAA** | **CGTCTCCGGGATTCCCGAGC** |
| ***Ccl2*** | **GGCTCAGCCAGATGCAGTTAA** | **CCTACTCATTGGGATCATCTTGCT** | **CCCCACTCACCTGCTGCTACTCATTCA** |
| ***Ces1f*** | **TGAGGCCAATTACTGAGCAAATT** | **GCGCATGCAGTGAACAATG** | **CTGGCTGTAAGACCACCACGTCTGCC** |
| ***Gpat3*** | **TGAAATCGGAGGAACCATCTATC** | **CGAAGCAGGTAGCTCACCAAGT** | **CCATAAAGTATAACCCCCAGTTCGGCGA** |
| ***Gpat4*** | **TGACTGAGCATGTCCAGGATAAAA** | **AAAGCTTCCCTTCTTGAACATCA** | **TTCCCAGAAGGAACCTGCATCAATAACACA** |
| ***Gsta3*** | **GGGCTGATATTGCCCTGGTT** | **TCTGCTTCTCAGCGCTTTCA** | **TCCTCTACCATGTGGAAGAGCTGGACCC** |
| ***Hmox1*** | **CACTTCGTCAGAGGCCTGCTA** | **GTCTGGGATGAGCTAGTGCTGAT** | **TGCAAGATACTGCCCCTGCAGAGACAC** |
| ***Itgam*** | **GGGTCATTCGCTACGTAATTGG** | **TGTTCACCAGCTGGCTTAGATG** | **AACCACAGTCCCGCAGAGAGCTCGA** |
| ***Itgax*** | **CCACTGTCTGCCTTCATATTCATG** | **AAGATGGCCCGGGTACTCA** | **TTGACCTGGCTCTAGACCATGGCCG** |
| ***Lbp*** | **CCGGCATCCGGTTGAA** | **TTCATGTCGGGATACTTTTTGTATATCT** | **ACCAAGGCCTTCCGTCCCTTCACTC** |
| ***Lipf*** | **CAGAAAACGCCTCCCTACTATGA** | **TGGCCACCGTTCCACACT** | **TGTCAGCCATGACCGTGCCAATTG** |
| ***Lrg1*** | **ATGGTCCCACCGAGTTTCC** | **GGAGAATTCCACCGACAGATG** | **AGCTCCCTCCCTGCCGACACTGT** |
| ***Mest*** | **AAGCCATGTAAAAGCACAACTATCTC** | **CCTACAAAGGCCTACGCATCTT** | **TTCCGACCACACCGACAGAATCTTGG** |
| ***Mrap*** | **TGGACGAGAAGAAGCTGAAAGC** | **AAAGAGGAGCACCACGAAGGTA** | **ACAAGCATTCCATTGTCATCGCCCTG** |
| ***Mrc2*** | **GAGCGACGGTCTAGGGTTTTC** | **GCCTCGGATATCATCGTCATC** | **TACCACAATTTTGCCCGGAGCCG** |
| ***Negr1*** | **AACAGATGATGGCCCGTACA** | **CAAACAAGTAAGGGTGACATTGG** | **CATCTCACTGTGCAAGTCCCACCGA** |
| ***Nkd1*** | **TTCACTCTATATGACTTCGACAACAATG** | **AGGAGTCAACCACTTCATAGATGGT** | **AAGTGACCCGTGAGGACATTACCAGCTTG** |
| ***Plagl1*** | **CTCCAAGTATAAGCTGATGAGACACAT** | **ACACGCGTAGGAGATCTTGTTG** | **TGAAGAACCACCTCCAGACCCACGATC** |
| ***Pparg2*** | **GCCTATGAGCACTTCACAAGAAATT** | **TGCGAGTGGTCTTCCATCAC** | **TCTGGCCCACCAACTTCGGAATCAG** |
| ***Ppib*** | **GGTGGAGAGCACCAAGACAGA** | **GCCGGAGTCGACAATGATG** | **ATCCTTCAGTGGCTTGTCCCGGCT** |
| ***Serpine1*** | **GGCACAGTGGCGTCTTCCT** | **GCCGAACCACAAAGAGAAAGG** | **CATCTCAGCCCGCATGGCCC** |
| ***Sfrp5*** | **CCAAGATCTGTGCCCAGTGT** | **TGCGCATCTTGACCACAAA** | **ATGGCCTCATGGAACAGATGTGCTCC** |
| ***Slc5a7*** | **CATATGCATAGGAGCTATTGGAGCTT** | **GAATCATGTCTGCTTCCTCCTTAGT** | **CACAGACTGGAACCAGACTGCCTACGG** |
| ***Tbp*** | **CTTCGTGCAAGAAATGCTGAAT** | **CAGTTGTCCGTGGCTCTCTTATT** | **TCCCAAGCGATTTGCTGCAGTCATC** |
| ***Tfr2*** | **GTACAACGTGCGCATCATGAG** | **GGAAAATGTGGCGGAATGG** | **TCCCAGTATGTGTCGCCAGCCGA** |
| ***Tph2*** | **TGGCTACAGGGAAGACAACGT** | **CAGCCACTGGTCTCACTGTGAA** | **CCGCAACTGGAAGACGTCTCCATGT** |
| ***Ucp2*** | **GCGTTCTGGGTACCATCCTAAC** | **GCGACCAGCCCATTGTAGA** | **CGCACTGAGGGTCCACGCAGC** |
